# Supplementary material for: The potential of gene therapy to rescue telomeric and extratelomeric defects in X-linked dyskeratosis congenita
Source: Mol Ther Adv. 2026 Jul 10;34(3):201811. doi: 10.1016/j.omta.2026.201811 (PMC13444285; doi:10.1016/j.omta.2026.201811)
Supplement: Supplementary file 1 [file mmc1.pdf]

OMTA, Volume *34*

## Supplemental information

# The potential of gene therapy to rescue telomeric and extratelomeric defects in X-linked dyskeratosis congenita

**Diego Llorente-Arroyo, María Luz Lozano, Susana Navarro, Leandro Sastre, Rosario Perona, Juan Antonio Bueren, Guillermo Guenechea, and Nestor Wenceslao Meza**

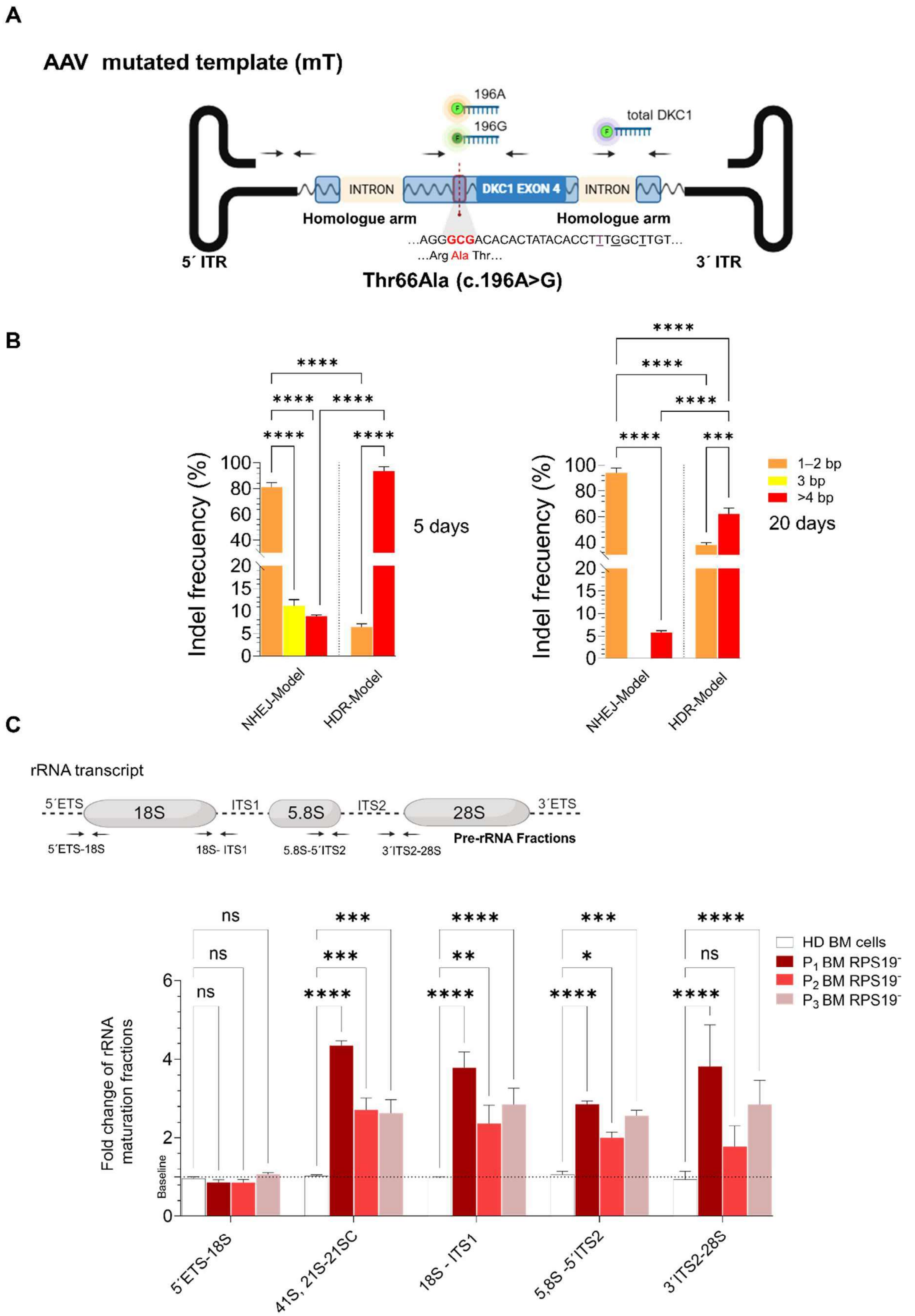
A
AAV mutated template (mT)
196A
196G
total DKC1
INTRON
DKC1 EXON 4
INTRON
Homologue arm
Homologue arm
…AGGGCGACACACTATACACCTTTGGCTTGT…
…Arg Ala Thr…
5´ ITR
3´ ITR
Thr66Ala (c.196A>G)
B
Indel frecuency (%)
5 days
20 days
NHEJ-Model
HDR-Model
1–2 bp
3 bp
>4 bp
C
rRNA transcript
5´ETS
18S
ITS1
5.8S
ITS2
28S
3´ETS
5´ETS-18S
18S- ITS1
5.8S-5´ITS2
3´ITS2-28S
Pre-rRNA Fractions
Fold change of rRNA maturation fractions
Baseline
HD BM cells
P1 BM RPS19-
P2 BM RPS19-
P3 BM RPS19-
5´ETS-18S
41S, 21S-21SC
18S - ITS1
5.8S -5´ITS2
3´ITS2-28S

**Fig. S1. Generation and consequences of a hypomorphic *DKC1* mutation in human HSPCs**

(A) Schematic representation of the single-stranded AAV (ssAAV) donor used to introduce the missense 196A>G (Thr66Ala) mutation into human $CD34^+$ cells by homology-directed repair (HDR). Arrows indicate the positions of flanking primers used to quantify modifications within *DKC1* exon 4 by PCR. The region containing the qPCR detection probes is indicated by coloured lines (see Supplementary Tables 2 and 3 for oligonucleotide sequences). (B) Distribution of indel mutations detected after editing of *DKC1* exon 4 in the NHEJ and HDR models. Orange bars represent frameshift indels of 1-2 base pairs (non-multiples of three) predicted to generate premature stop codons. Yellow bars indicate in-frame deletions of exactly three base pairs (corresponding to the loss of one amino acid). Red bars represent larger deletions (>4 base pairs), which were grouped for clarity. For comparisons of indel frequencies between editing models, Tukey's multiple-comparison correction was applied. (C) Ribosomal RNA (rRNA) maturation analysis in edited cells assessed by RT-qPCR. The schematic at the top illustrates the immature rRNA regions recognized by each primer pair, corresponding to four distinct pre-rRNA fractions that capture successive stages of rRNA processing. A fifth fraction (41S/21S-21SC) was calculated by subtracting the 5′ETS-18S signal from the 18S-ITS1 signal, thereby reflecting an intermediate-to-late stage of rRNA maturation (see Methods and Supplementary Table 1). The graph shows the relative abundance of rRNA precursors in bone marrow samples from patients with Diamond–Blackfan anemia (P1–P3; all RPS19-deficient), used as positive controls for the rRNA maturation assay. N = 3 patients, with each sample measured in

technical triplicates. Data are shown as mean ± SEM. Statistical analysis was performed using Fisher's least significant difference (LSD) test.

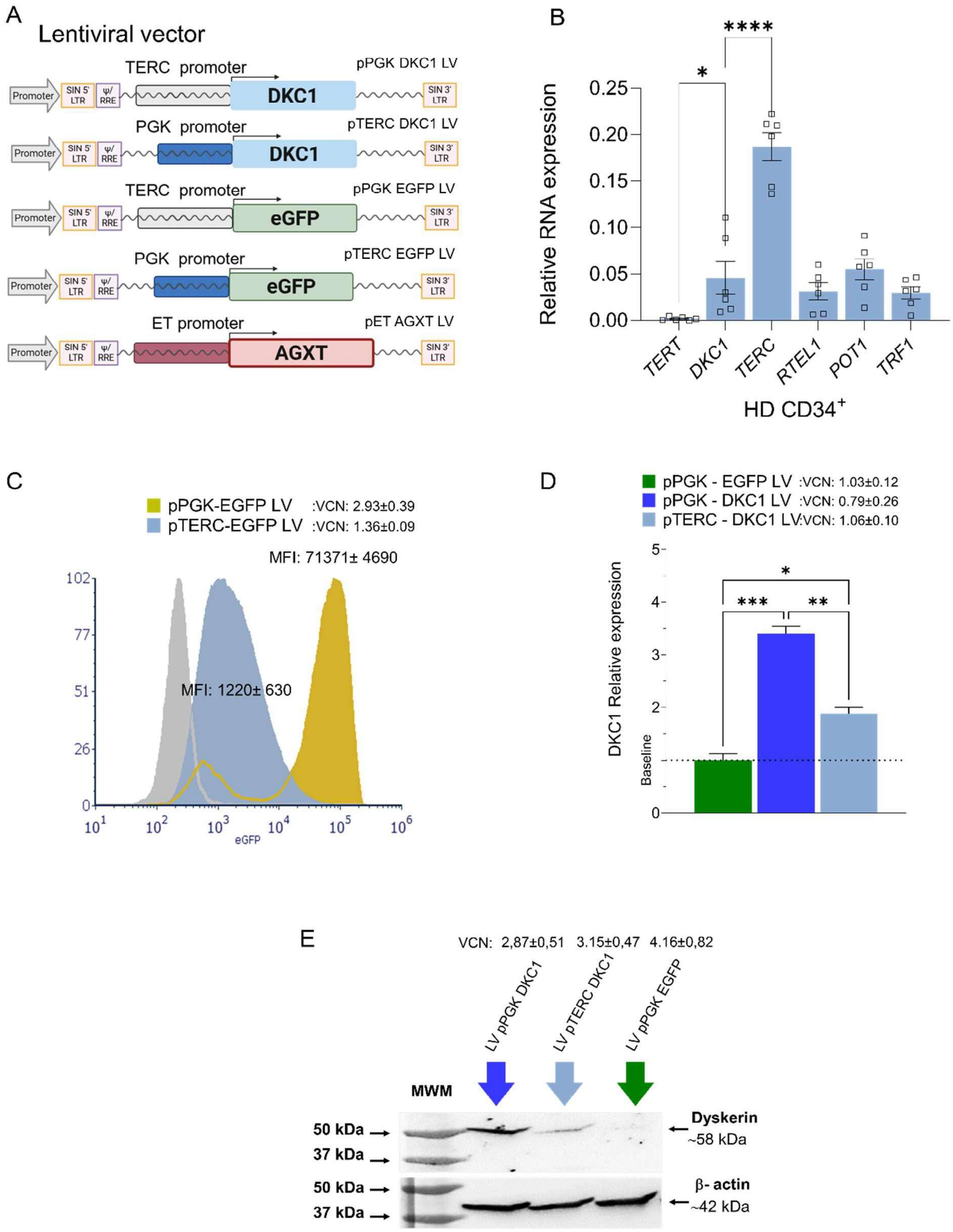
A
Lentiviral vector
TERC promoter
pPGK DKC1 LV
DKC1
PGK promoter
pTERC DKC1 LV
TERC promoter
pPGK EGFP LV
eGFP
pTERC EGFP LV
ET promoter
pET AGXT LV
AGXT
Promoter
SIN 5' LTR
ψ/ RRE
SIN 3' LTR
B
Relative RNA expression
TERT
DKC1
TERC
RTEL1
POT1
TRF1
HD CD34+
C
pPGK-EGFP LV :VCN: 2.93±0.39
pTERC-EGFP LV :VCN: 1.36±0.09
MFI: 71371± 4690
MFI: 1220± 630
eGFP
D
pPGK - EGFP LV :VCN: 1.03±0.12
pPGK - DKC1 LV :VCN: 0.79±0.26
pTERC - DKC1 LV:VCN: 1.06±0.10
DKC1 Relative expression
Baseline
E
VCN: 2,87±0,51 3.15±0,47 4.16±0,82
LV pPGK DKC1
LV pTERC DKC1
LV pPGK EGFP
MWM
50 kDa
37 kDa
Dyskerin
~58 kDa
β- actin
~42 kDa

**Fig. S2. Functional characterization of *DKC1* lentiviral vectors**

(A) Schematic representation of the lentiviral vectors used to express human *DKC1*. Therapeutic vectors encoding dyskerin cDNA were constructed using either the human phosphoglycerate kinase promoter (pPGK) or a 798-bp fragment of the endogenous human *TERC* promoter (pTERC). Corresponding control vectors expressed EGFP instead of *DKC1*, and an unrelated liver-specific lentiviral vector (pET-AGXT) was used as a negative control during *in vivo* experiments (see Methods for details). (B) Relative abundance of *TERC*, *DKC1*, *TERT*, *RTEL1*, *POT1*, and *TRF1* transcripts in healthy donor $CD34^+$ cells five days after lentiviral transduction, normalized to the *GAPDH* housekeeping gene. Data represent mean ± SEM from six independent experiments (N = 6). Statistical significance was assessed using Tukey's multiple-comparison test. (C) Mean fluorescence intensity (MFI) of EGFP in healthy donor $CD34^+$ cells 20 days after transduction with a high multiplicity of infection (MOI = 100) of the promoter test vectors pPGK-EGFP (dark yellow) or pTERC-EGFP (light blue). This panel reflects the relative transcriptional strength of each promoter as assessed by EGFP expression. (D) Relative dyskerin mRNA levels in healthy donor $CD34^+$ cells 20 days after transduction with either the pPGK-DKC1 (dark blue) or pTERC-DKC1 (light blue) lentiviral vectors, normalized to levels measured in cells transduced with the control pPGK-EGFP vector (green). Data represent mean ± SEM from two independent experiments (N = 2). Tukey's multiple-comparison test was used for statistical analysis. (E) Western blot analysis of dyskerin protein expression driven by the pPGK-DKC1 and pTERC-DKC1 lentiviral vectors in transduced NIH-3T3 cells. A band of approximately 58 kDa corresponding to human dyskerin is detected in cells transduced with either *DKC1* vector and is absent in untransduced controls. The vector copy number per cell (VCN/cell) measured for

each condition is indicated in panels C–E, demonstrating comparable transduction levels across experimental groups.

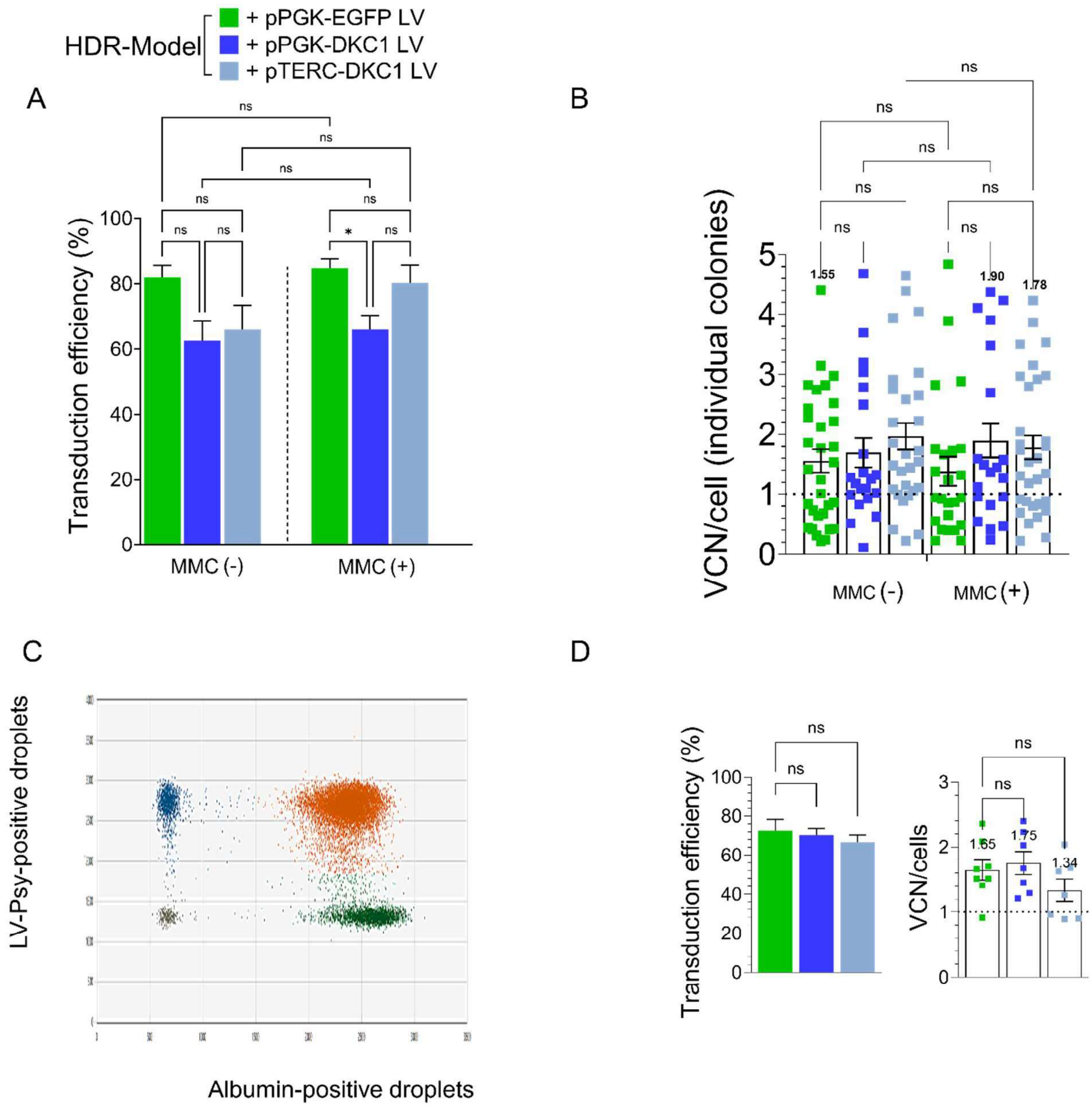


**Fig. S3. Lentiviral transduction efficiency in X-DC-like HSPCs**

(A) Percentage of hematopoietic colonies positive for lentiviral proviral integration across the experimental conditions shown in Fig. 3. Data represent mean ± SEM from four independent experiments (N = 4). (B) Vector copy number per cell (VCN/cell) measured in individual colonies derived from the experiments shown in Fig. 3. Each bar represents the

mean VCN calculated from 20-40 colonies per condition, illustrating the distribution of lentiviral integration events. (C) Two-dimensional representation of VCN in bulk cultures, measured by droplet digital PCR (ddPCR) after 20 days of standard liquid culture for each experimental group. (D) Lentiviral transduction efficiency and vector copy number in 20-day liquid cultures corresponding to the experimental conditions shown in Fig. 4. Left: percentage of lentivirus-transduced cells (transduction rate). Right: corresponding VCN per cell measured in bulk culture. These paired analyses allow comparison of transduction efficiency and vector copy stability over time. Statistical comparisons between groups in panel D were performed using Tukey's multiple-comparison test.

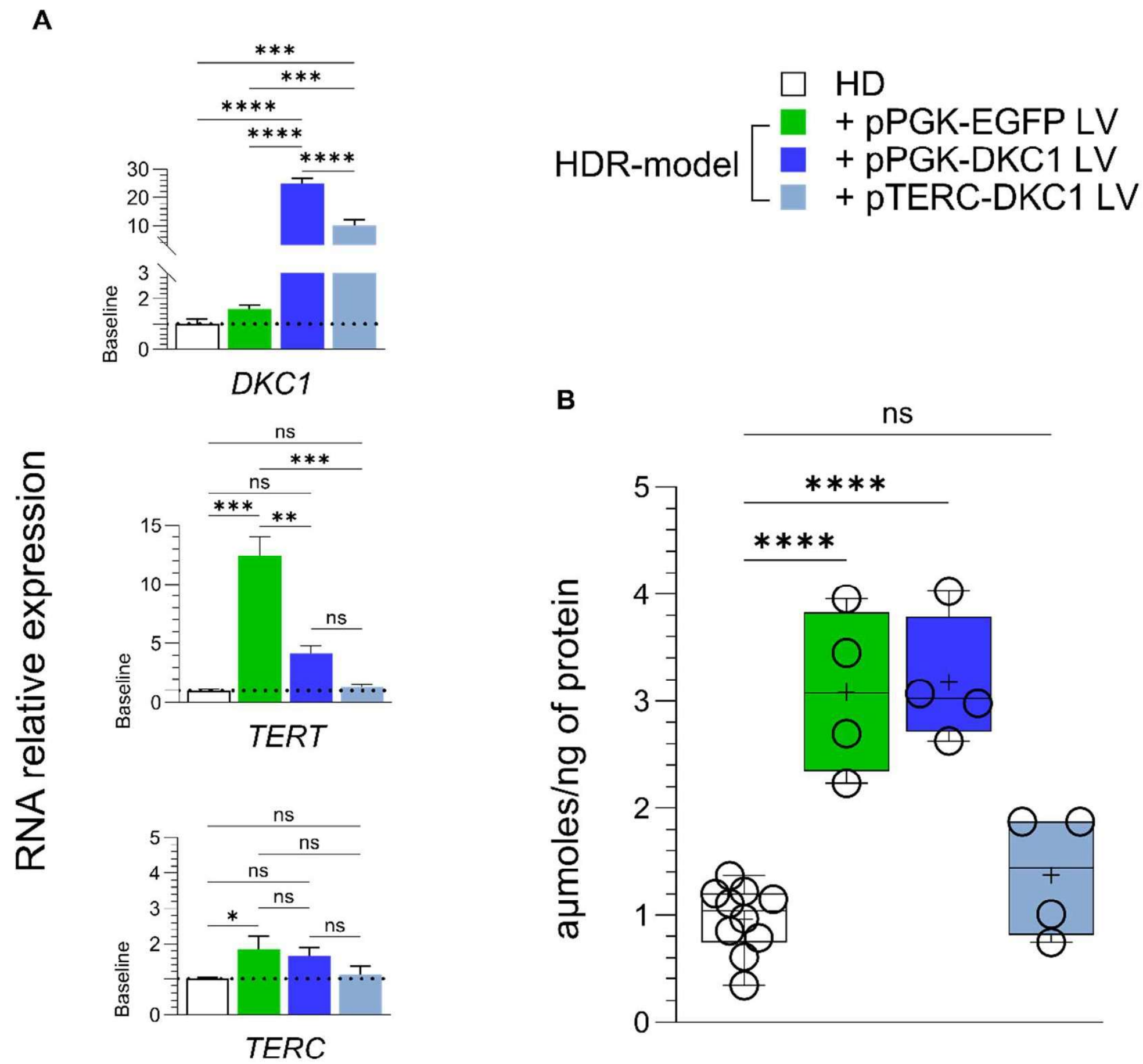


**Fig. S4. Regulation of telomerase-related components in X-DC-like HSPCs after *DKC1* gene transfer.** (A) Expression levels of *DKC1*, *TERT*, and *TERC* transcripts in X-DC–like CD34$^+$ cells transduced with the indicated lentiviral vectors: pPGK-EGFP (green bars; non-therapeutic control), pPGK-DKC1 (dark blue bars), or pTERC-DKC1 (light blue bars). Unedited and untransduced CD34$^+$ cells from healthy donors (HD; white bars) were cultured in parallel and used as baseline controls. RNA levels were quantified five days post-transduction, at a time point when cultures contained approximately 30–60% CD34$^+$ progenitor cells. Data represent mean ± SEM from three independent experiments (N = 3). (B) Telomerase activity measured in cell lysates from the same experimental groups shown

in panel A, expressed as units of telomere repeat amplification protocol (TRAP) product per nanogram of protein. Sample sizes were N = 4 for each transduced group and N = 8 for the untreated HD control group. Statistical comparisons between groups were performed using Fisher's least significant difference (LSD) multiple-comparison test.

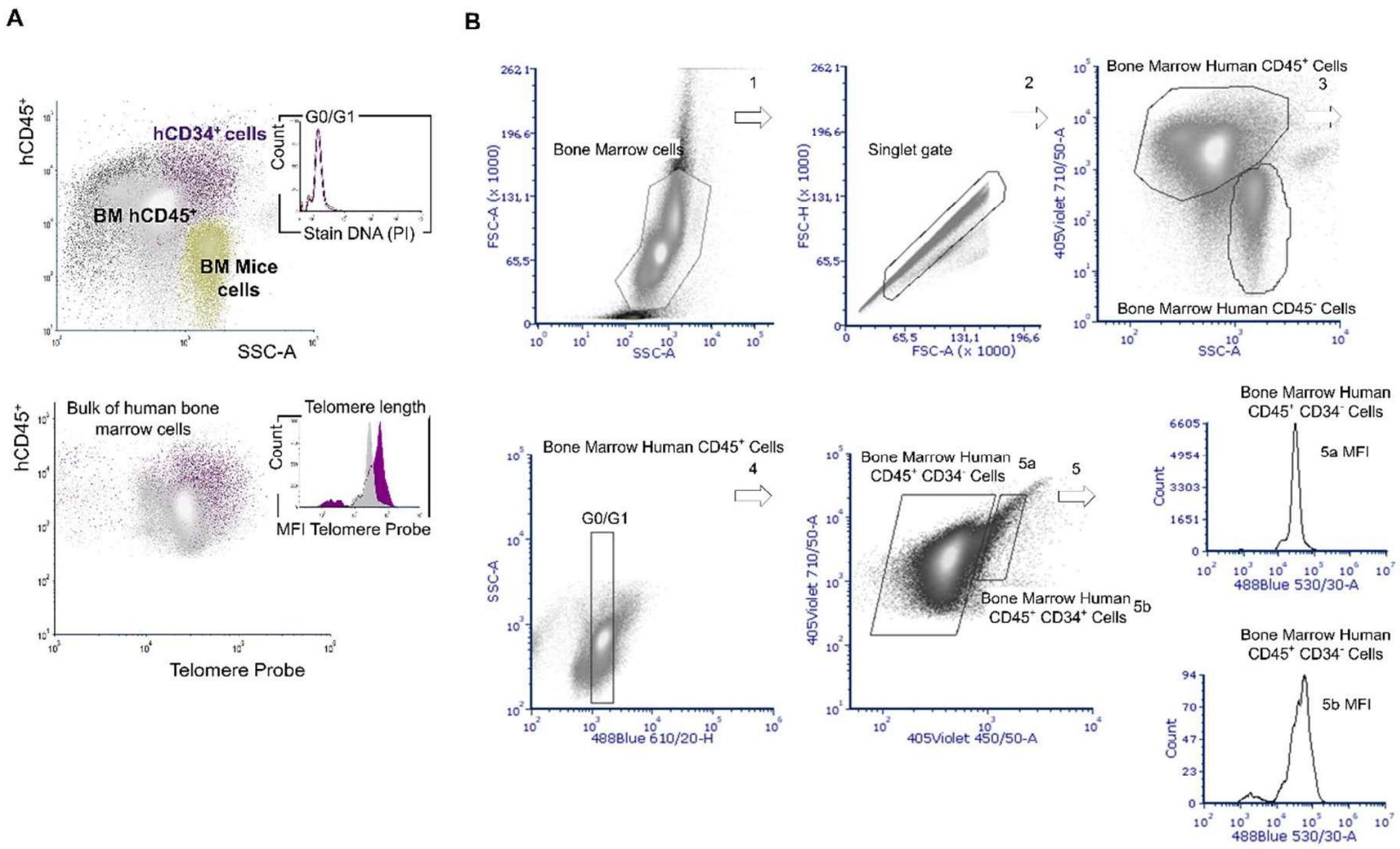


**Fig. S5. Flow-FISH gating strategy for telomere length analysis of engrafted human hematopoietic cells in mouse bone marrow.** (A) Flow-FISH analysis of telomere length in engrafted human cells (representative dot plots). Top: Flow cytometry gating of human and mouse cells, showing human $CD45^+$ cells (grey), human $CD45^+CD34^+$ cells (purple), and residual mouse cells (ochre) on an hCD45 versus side scatter (SSC) plot. $G_0/G_1$ cell-cycle phases are indicated for the human cell populations. Bottom: Telomeric probe fluorescence profiles in human $CD45^+CD34^-$ and $CD45^+CD34^+$ cells, shown as overlays of mean fluorescence intensity (MFI). (B) Gating strategy used for Flow-FISH–based telomere length assessment in human hematopoietic cells engrafted in NSG mice. Sequential gating steps are indicated by numbers in the dot plots. Human cells were identified using CD45 and CD34 monoclonal antibodies conjugated to Brilliant Violet 711 and Brilliant Violet 421, respectively. $G_0/G_1$ cell-cycle phases were discriminated using propidium iodide as a DNA

content marker. Following gating, telomere probe MFI was quantified in the $CD45^+CD34^-$ (population 5a) and $CD45^+CD34^+$ (population 5b) compartments, as shown in dot plot 5.

**Table S1. Pre-rRNA Species Detected by Specific Maturation Amplicons**

| Pre-rRNA fraction (amplicons): | Northern Blot detected pre-rRNA species | | | | | | | | | | | |
|---|---|---|---|---|---|---|---|---|---|---|---|---|
| | 47S | 45S | 43S | 41S | 34S | 32S | 30S | 26S | 21S | 21S-C | 18S-E | 12S |
| 5´ETS-18S | X | X | X | | X | | X | X | | | | |
| 18S- ITS1 | X | X | X | X | X | | X | X | X | X | | |
| 5,8S - 5´ITS2 | X | X | X | X | | X | | | | | | X |
| 3´ITS2-28S | X | X | X | X | | X | | | | | | |
| *41S, 21S-21SC | | | | X* | | | | | X* | X* | | |

*41S, 21S-21SC fraction is calculated as the difference between fractions (18S- ITS1)- 5´ETS-18S.

**Table S2: Description of the oligonucleotides used in the study**

| Oligonucleotide | Sequence (5' → 3') | Detection |
|---|---|---|
| Psi Fw | CAGGACTCGGCTTGCTGAAG | Psi |
| Psi Rv | TCCCCCGCTTAATACTGACG | Psi |
| Alb Fw | GCTGTCATCTCTTGTGGGCTG | Albumin |
| Alb Rv | ACTCATGGGAGCTGCTGGTTC | Albumin |
| seqDKC1Fw | GATGTAGCCGTGAGTAGTAATGTGTTTG | Genomic human DKC1 |
| seqDKC1Rw | CAATCCCCACATACTCTTTGCCTG | Genomic human DKC1 |
| DKC1 Model Fw | TGTTTTGTGTTGTGTCGTTGCTT | DKC1 intron 3 |
| DKC1 Model Rv | TTGCACAGTGCCTCCTCCC | DKC1 intron 4 |
| DKC1 Control Fw | TTCCAGGACAGGTTTCATTAATCTT | DKC1 intron 4-exon5 |
| DKC1 Control Rv | CCGAAGTATCCGTCGAATCC | DKC1 exon 5 |
| HBB Fw | GCTTCTGACACAACTGTGTTCACTAGC | HBB |
| HBB Rv | CACCAACTTCATCCACGTTCACC | HBB |
| Telomere Fw | CGGTTTGTTTGGGTTTGGGTTTGGGTTTGGGTT TGGGTT | Telomere |
| Telomere Rv | GGCTTGCCTTACCCTTACCCTTACCCTTACCCT TACCCT | Telomere |
| DKC1 Fw | AAGCCCCGCAGGTAGTTGCC | DKC1 cDNA |
| DKC1 Rv | CTCGGCCCCGCTCTCCAGAC | DKC1 cDNA |
| TERC Fw | GCGCGCTGTTTTTCTCGCTGAC | TERC cDNA |
| TERC Rv | TAGAATGAACGGTGGAAGGCGGC | TERC cDNA |
| TERT Fw | AACATGGACTACGTCGTGGGAGC | TERT cDNA |
| TERT Rv | ACGCTGAACAGTGCCTTCACCC | TERT cDNA |
| GAPDH Fw | AACGGATTTGGTCGTATTGG | GAPDH cDNA |
| GAPDH Rv | TGGGTGGAATCATATTGGAAC | GAPDH cDNA |
| CDKN1A Fw | GCTCTGCTGCAGGGGACAGC | CDKN1A cDNA |
| CDKN1A Rv | TCAGCCGGCGTTTGGAGTGG | CDKN1A cDNA |
| MYC Fw | TGCTCCATGAGGAGACACCGC | MYC cDNA |
| MYC Rv | TTGCCAGGAGCCTGCCTCTTTTC | MYC cDNA |
| RTEL1 Fw | CCGCGGGCTGTAGCCAACTC | RTEL1 cDNA |
| RTEL1 Rv | TCTCCTCCTGGGGTCCCGGT | RTEL1 cDNA |
| POT1 Fw | AGGAGACTCAGGGTATCACCAGC | POT cDNA |
| POT1 Rv | AGTGCGAGGTATGATAGGGGCTC | POT cDNA |

| | | |
|---|---|---|
| CEBPA Fw | CAAGAGCCGCGACAAGGCCA | CEBPA cDNA |
| CEBPA Rv | GAAGATGCCCCGCAGCGTGT | CEBPA cDNA |
| TRF1 Fw | ACCTGTTTCAAAGAGTCAGCCGGT | TRF1 cDNA |
| TRF1 Rv | CCCTCTCCATATTTCCTCACGCCA | TRF1 cDNA |
| TRF2 Fw | AGGAGACTTGGGTGGAAGAGGATGA | TRF2 cDNA |
| TRF2 Rv | TCCAGCCTTGACCCACTCGCT | TRF2 cDNA |
| CCNB1 Fw | TGGTGCCAGTGCCAGTGTCTG | CCNB1 cDNA |
| CCNB1 Rv | TTCCATTGGGCTTGGAGAGGCAG | CCNB1 cDNA |
| CDK2 Fw | TCATCGGGTCCTCCACCGAGAC | CDK2 cDNA |
| CDK2 Rv | ACCACAGGGTCACCACCTCATGG | CDK2 cDNA |
| TZAP Fw | GGAACAGGTCTTCACGTGCTCTG | TZAP cDNA |
| TZAP Rv | GCAGGAGGAACACTTGTAGGGC | TZAP cDNA |
| 5´ETS-18S Fw | CGTCCGTCCGTCGTCCTCCT | 5´ETS rRNA |
| 5´ETS-18S Rv | ACTGGCAGGATCAACCAGGTAGGTA | 18S rRNA |
| 18S-ITS1 Fw | AGGATCATTAACGGAGCCCGGAGG | 18S rRNA |
| 18S-ITS1 Rv | GAGCGAACGAACGGGCACGC | ITS1 rRNA |
| 18S Fw | GTGACCACGGGTGACGGGGA | 18S rRNA |
| 18S Rv | TACCTCCCCGGGTCGGGAGT | 18S rRNA |
| 5,8S Fw | GCGGTGGATCACTCGGCTCG | 5,8S rRNA |
| 5,8S Rv | CGTAGCCCCGGGAGGAACCC | 5,8S rRNA |
| 5,8S-5'ITS2 Fw | TGTCTGAGCGTCGCTTGCCG | 5,8S rRNA |
| 5,8S-5'ITS2 Rv | TGCGCTTAGGGGGACGGAGG | 5'ITS2 rRNA |
| 3'ITS2-28S Fw | TGTCTGAGCGTCGCTTGCCG | 3'ITS2 rRNA |
| 3'ITS2-28S Rv | TGAGGTCGCGTCTCGGAGGG | 28S rRNA |
| 28S Fw | TGCGGACCAGGGGAATCCGA | 28S rRNA |
| 28S Rv | CGCCGTTTACCCGCGCTTCA | 28S rRNA |
| GAPDH Fw | CCCCTCTGCTGATGCCCCCA | GAPDH cDNA |
| GAPDH Rv | TGACCTTGGCCAGGGGTGCT | GAPDH cDNA |

Oligonucleotides used in this study (sequences are given 5′→3′). Each oligonucleotide is listed with its sequence and the target or purpose for which it was designed (e.g., *Psi*, *Albumin*, *DKC1* genomic locus, *HBB*, etc.).

**Table S3. Description of the different taqman probes used in the study amplicons**

| Taqman probe | sequence | Fluorochrome/quencher |
|---|---|---|
| Psi | CGCACGGCAAGAGGCGAGG | FAM/BHQ1 |
| Alb | CCTGTCATGCCCACACAAATCTCTCC | VIC/BHQ1 |
| DKC1WT probe | ACAACACACTATACACCTCTTGC | HEX/BHQ1 |
| DKC1X-DC probe | AGGGCGACACACTATACACCT | FAM/BHQ1 |
| DKC1Control probe | ACAAGCCCTCTAACCCCTCTTCC | 5Cy5/BHQ2 |

Sequences and labels of TaqMan probes used in this study. Each probe's DNA sequence is shown along with its fluorescent reporter and quencher (e.g., FAM/BHQ1, VIC/BHQ1, HEX/BHQ1, Cy5/BHQ2), corresponding to the specific qPCR/ddPCR amplicons in the assay.
