# Supplementary material for: The potential of gene therapy to rescue telomeric and extratelomeric defects in X-linked dyskeratosis congenita
Source: Mol Ther Adv. 2026 Jul 10;34(3):201811. doi: 10.1016/j.omta.2026.201811 (PMC13444285; doi:10.1016/j.omta.2026.201811)
Supplement: Supplementary file 2 [file mmc2.pdf]

Molecular Therapy
Advances
Original Article

The potential of gene therapy to rescue telomeric and extratelomeric defects in X-linked dyskeratosis congenita

Diego Llorente-Arroyo,[1] María Luz Lozano,[1] Susana Navarro,[1] Leandro Sastre,[2] Rosario Perona,[2] Juan Antonio Bueren,[1] Guillermo Guenechea,[1] and Nestor Wenceslao Meza[1]

[1]Division of Hematopoietic Innovative Therapies, Biomedical Innovation Unit, Centro de Investigaciones Energéticas Medioambientales y Tecnológicas (CIEMAT), Centro de Investigación Biomédica en Red de Enfermedades Raras (CIBERER) and Advanced Therapies Unit - Instituto de Investigación Sanitaria Fundación Jiménez Díaz (IIS-FJD/UAM), 28040 Madrid, Spain; [2]Instituto de Investigaciones Biomédicas "Alberto Sols" (CSIC/UAM) and Centro de Investigación Biomédica en Red de Enfermedades Raras (CIBERER), 28029 Madrid, Spain

Dyskeratosis congenita (DC) is a rare inherited disorder characterized by bone marrow failure, pulmonary fibrosis, and cancer predisposition. X-linked DC (X-DC) is caused by mutations in the *DKC1* gene, which encodes dyskerin, a multifunctional protein that is essential for telomere maintenance and multiple RNA-associated processes. Here, we first generated human X-DC HSPC models by introducing *DKC1* frameshift mutations or the hypomorphic c.196 A>G mutation, into cord blood CD34$^+$ cells from healthy male donors. These X-DC-like HSPCs recapitulated key hematopoietic features of the disease, including impaired proliferation, defective telomere maintenance and altered rRNA maturation. To correct these defects, lentiviral vectors were constructed carrying *DKC1* under the PGK promoter or a weaker promoter driven by *TERC* regulatory sequences. While PGK-driven expression of dyskerin only restored the proliferation of X-DC-like HSPCs, pTERC-mediated expression of dyskerin also corrected the telomere maintenance and rRNA maturation defects characteristic of X-DC cells, without altering the functional properties of healthy HSPCs. These findings indicate that *TERC* promoter-driven dyskerin expression can functionally rescue key telomeric and extratelomeric defects in HSPCs carrying frameshift or hypomorphic *DKC1* mutations, supporting further preclinical development of this strategy and providing a rationale for its evaluation as a therapeutic approach for X-linked dyskeratosis congenita.

INTRODUCTION

Telomere biology disorders (TBDs) constitute a heterogeneous group of rare diseases caused by germline mutations in genes involved in the telomere maintenance mechanisms (TMMs).[1,2] The primary function of TMM genes is the preservation of telomere length in highly proliferative cells.[1,3] Nevertheless, non-canonical extratelomeric functions, such as those related to gene expression, RNA processing, signal transduction, and other molecular and cellular processes have also been reported for TMM genes.[2,4–8]

The archetype of TBDs is dyskeratosis congenita (DC), a rare inherited syndrome that leads to bone marrow failure (BMF) in approximately 80% of patients during their childhood or adolescent years.[4,9] Patients with DC are also at elevated risk of pulmonary fibrosis, liver disease, and cancer (including squamous cell carcinoma and hematological malignancies), as well as other medical complications.[4,6,10,11] Høyeraal-Hreidarsson syndrome represents one of the most severe forms of DC, with patients experiencing failure of multiple organ systems within the first decade of life.[12–14] A common feature of patients with DC is the presence of short telomeres, which serve as predictors of early-onset injury in tissues with high proliferation and renewal rates, such as the epithelial and hematopoietic systems.[4,15] DC is associated with mutations in any of the 18 TMM-related genes identified to date. Among them, mutations in the *DKC1*, *TERT*, *TERC*, and *TINF2* genes account for approximately 50% of reported cases of DC.[1,4,5,16]

Received 21 January 2026; accepted 8 July 2026;
https://doi.org/10.1016/j.omta.2026.201811.

Correspondence: Juan Antonio Bueren, Division of Hematopoietic Innovative Therapies, Biomedical Innovation Unit, Centro de Investigaciones Energéticas Medioambientales y Tecnológicas (CIEMAT), Centro de Investigación Biomédica en Red de Enfermedades Raras (CIBERER) and Advanced Therapies Unit - Instituto de Investigación Sanitaria Fundación Jiménez Díaz (IIS-FJD/UAM), 28040 Madrid, Spain.
E-mail: juan.bueren@ciemat.es
Correspondence: Guillermo Guenechea, Division of Hematopoietic Innovative Therapies, Biomedical Innovation Unit, Centro de Investigaciones Energéticas Medioambientales y Tecnológicas (CIEMAT), Centro de Investigación Biomédica en Red de Enfermedades Raras (CIBERER) and Advanced Therapies Unit - Instituto de Investigación Sanitaria Fundación Jiménez Díaz (IIS-FJD/UAM), 28040 Madrid, Spain.
E-mail: g.guenetxea@ciemat.es
Correspondence: Nestor Wenceslao Meza, Division of Hematopoietic Innovative Therapies, Biomedical Innovation Unit, Centro de Investigaciones Energéticas Medioambientales y Tecnológicas (CIEMAT), Centro de Investigación Biomédica en Red de Enfermedades Raras (CIBERER) and Advanced Therapies Unit - Instituto de Investigación Sanitaria Fundación Jiménez Díaz (IIS-FJD/UAM), 28040 Madrid, Spain.
E-mail: nestorw.meza@ciemat.es

© 2026 The Authors. Published by Elsevier Inc. on behalf of The American Society of Gene and Cell Therapy. This is an open access article under the CC BY license (http://creativecommons.org/licenses/by/4.0/).

Germline mutations in *DKC1* result in inherited X-linked recessive DC (X-DC), which accounts for up to 25% of all DC cases.[17] In over 90% of X-DC patients, *DKC1* missense mutations have been identified.[18,19] *DKC1* encodes the enzyme dyskerin pseudouridine synthase 1. This enzyme associates with multiple H/ACA motif-containing RNAs, including human telomerase non-coding RNA (TERC) in the telomerase ribonucleoprotein (RNP) complex. Within these RNP complexes, dyskerin plays a fundamental role in telomere maintenance and ribosomal RNA modification. Dyskerin is ubiquitously expressed and promotes pseudouridylation and post-transcriptional processing of a wide range of RNAs, which affect their stability, structure, and function. This pleiotropic influence on the processing of diverse RNA targets suggests a central role for these non-canonical dyskerin functions in the pathogenesis of the disease, in addition to the accelerated telomere shortening characteristic of patients with X-DC.[6,19,20]

As in other inherited BMF syndromes (e.g., Fanconi anemia [FA]), BMF constitutes the main cause of mortality in young DC patients.[21] While androgen therapy may offer a temporary hematological improvement, hematopoietic stem and progenitor cell (HSPC) transplantation from matched healthy donors remains the only potentially curative treatment for BMF in these patients.[22–25] Reported overall survival rates following HSPC transplantation are still modest, approximately 55% at 5 years.[26] Additionally, the allogeneic HSCT of these patients is associated with substantial morbidity largely driven by graft-versus-host disease (GVHD) and conditioning regimen-associated toxicity, which in turn contribute to serious organ complications and an increased long-term risk of malignancy.[26–28]

Gene therapy (GT) thus represents a promising therapeutic alternative for DC, in which the lessons learned from FA GT can be directly applied.[29,30] One key advantage of FA GT is that gene-corrected autologous HSPCs can engraft the patient in the absence of any conditioning regimen,[29–31] making this GT model particularly attractive for DC. In this study, we generated a clinically relevant model of X-DC by introducing loss-of-function or hypomorphic *DKC1* mutations into human HSPCs, which closely reproduced key HSPC defects previously observed in X-DC patients. Using this platform, we evaluated a lentiviral GT strategy in X-DC-like HSPCs and found that ectopic dyskerin expression driven by regulatory elements from the telomerase RNA component (*TERC*) efficiently restored disease-associated cellular phenotypes while leaving healthy cells unaffected. Importantly, these observations show that therapeutic efficacy depends on maintaining dyskerin expression within a functionally tolerated range, establishing dosage control as a central design principle for dyskerin-based therapy. Within this framework, our study provides preclinical evidence that GT can restore the molecular and cellular hallmarks of X-DC in human HSPCs.

RESULTS

The generation of *DKC1* indel and hypomorphic mutations in human HSPCs results in phenotypic defects characteristic of X-DC

In an initial set of experiments, we aimed to investigate the phenotypic defects associated with *DKC1* mutations in human HSPCs. Since X-DC is caused by mutations in the X-linked *DKC1* gene, umbilical cord blood (CB) $CD34^+$ cells from healthy male newborns were used as targets for two gene-editing approaches (see experimental approaches in Figure 1A). To generate indels potentially resulting in *DKC1* knockout (KO) mutations, $CD34^+$ cells were nucleofected with a sgRNA/Cas9 RNP complex that targets exon 4 of *DKC1* (non-homologous end joining [NHEJ] model). Additionally, to generate a *DKC1* hypomorphic mutation, CB $CD34^+$ cells were edited with the same RNP complex and then transduced with adeno-associated virus vectors (AAV) carrying a *DKC1* donor fragment with a missense mutation (Thr66Ala missense variant; c.196 A>G SNP at X:154765931) in exon 4 of *DKC1*(see Figure S1A; homology-directed repair [HDR] model) previously identified in X-DC patients.[13,19] Both *DKC1* indel and missense mutations are therefore present in these cells. Indels introduced by the RNP complex created premature termination codons downstream of mutated *DKC1* exon 4 (Figure S1B). Interestingly, the proportion of short indels generated in the NHEJ model was much higher compared with those identified in HDR-treated cells, suggesting that small deletions, rather than larger ones, were preferentially corrected by HDR in these cells (Figure S1B).

The proportion of *DKC1* mutations in edited $CD34^+$ cells was first evaluated using clonogenic assays, which allowed us to capture editing outcomes at the level of individual hematopoietic progenitors. As shown in Figure 1B (top), 47% of the hematopoietic colonies generated by NHEJ harbored *DKC1* indels. In the HDR experiments, 17% of the colonies carried *DKC1* indels while 60% of them carried the hypomorphic Thr66Ala mutation (Figure 1B, bottom). To investigate whether a cytotoxic stress modified the molecular and cellular hallmarks associated with *DKC1* mutations, edited $CD34^+$ cells were exposed to a low concentration (10 nM) of mitomycin C (MMC), a drug that generates cross-links in the DNA, including the telomeric DNA, as well as ribosomal RNA degradation.[32,33] In both editing models, MMC induced a significant reduction in the percentage of colonies harboring *DKC1* indels. However, this drug did not induce significant changes in the proportion of colonies carrying the hypomorphic mutation (Figure 1B).

When total colony numbers were evaluated (Figure 1C), we observed that colony numbers in the NHEJ model were significantly lower than those in the HD control and HDR groups. In contrast, colony numbers in the HDR and the HD groups were similar. Upon MMC challenge, total colony numbers in both *DKC1*-editing models were significantly lower compared with the HD group, with a more marked reduction in the NHEJ model (Figure 1C). Analysis of lineage distribution of colonies showed a relative enrichment of BFU-E

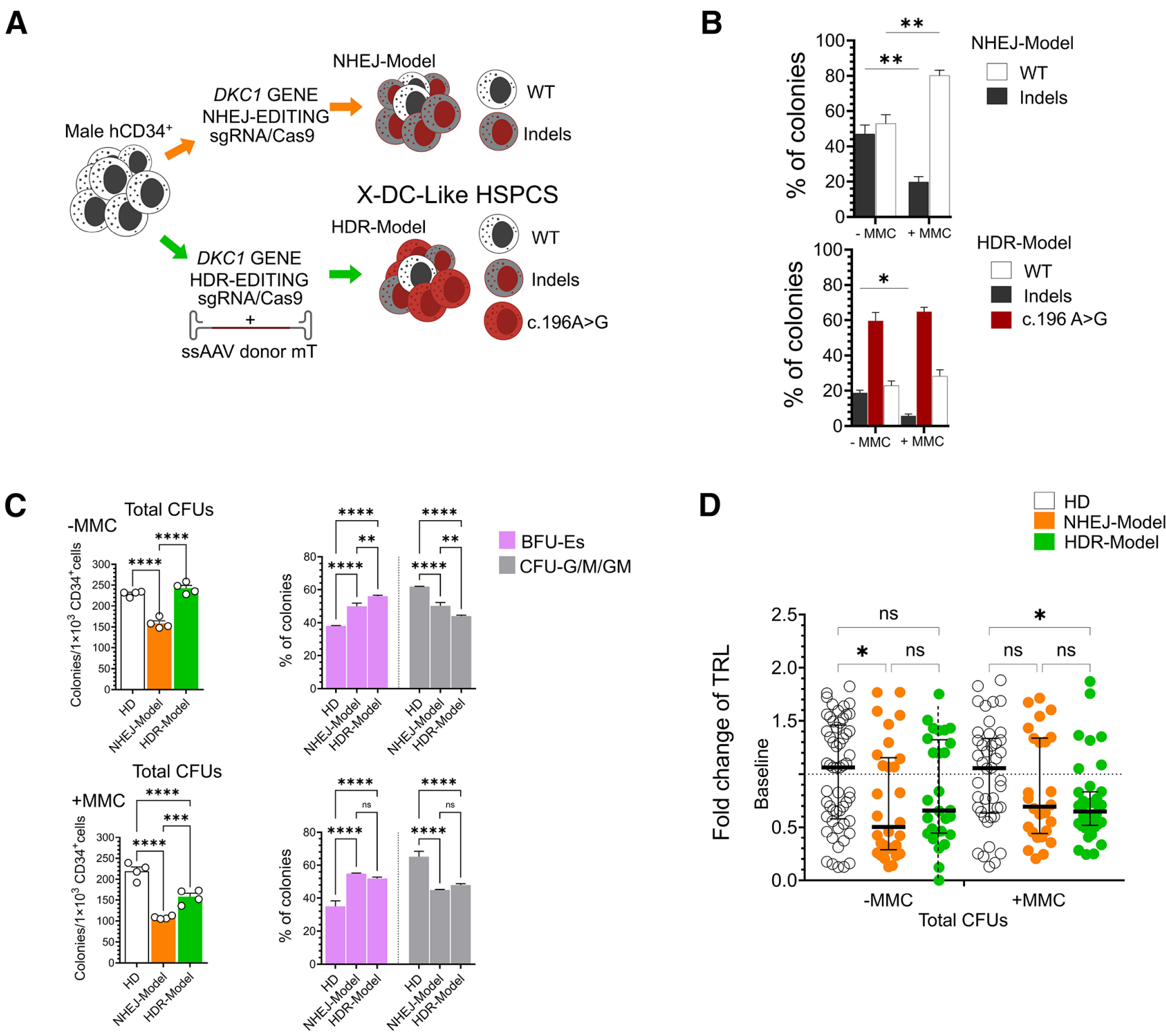

Figure 1. Clonogenic and telomere defects associated with frameshift and hypomorphic mutations in *DKC1*

(A) Schematic representation of the gene-editing strategies used to generate X-DC-like human HSPCs. Cord blood-derived CD34$^+$ cells from male donors were edited either by non-homologous end joining (NHEJ) to generate *DKC1* indels or by homology-directed repair (HDR) using an ssAAV donor template carrying the c.196 A>G (Thr66Ala) hypomorphic mutation. Because all experiments were performed in male cells, editing outcomes reflect modification of the single endogenous *DKC1* allele. (B) Distribution of *DKC1* genotypes in hematopoietic colonies generated from the NHEJ and HDR models in the absence or presence of mitomycin C (MMC). In the NHEJ model, colonies were classified as WT or indel-bearing. In the HDR model, colonies were classified as WT, indel-bearing, or c.196 A>G. Genotypes were determined in individual colonies by qPCR. Data represent mean ± SEM from 4 independent experiments. Bonferroni correction was applied for multiple comparisons. (C) Clonogenic output of DKC1-edited CD34$^+$ cells cultured in methylcellulose in the absence or presence of MMC. Left panels show total colony numbers. Right panels show the relative distribution of erythroid colonies (BFU-Es) and myeloid colonies (CFU-G/M/GM) in each condition. Data represent mean ± SEM from four independent experiments. Statistical analysis was performed using one-way ANOVA with Tukey post hoc test. (D) Telomere length in colonies derived from *DKC1*-edited CD34$^+$ cells relative to healthy donor (HD) colonies, measured by qPCR and expressed as fold change relative to HD baseline. Analyses are shown separately for colonies generated in the absence or presence of MMC. Only samples meeting the quality criteria described in materials and methods were included. Each dot represents an individual colony (n: 36–90); horizontal bars indicate mean ± SEM. Statistical analysis was performed using one-way ANOVA with Fisher's LSD post hoc test.

colonies compared to myeloid colonies, suggesting that dyskerin dysfunction may initially trigger an adaptive erythroid-biased response.

These results revealed differences in the severity of indel and hypomorphic *DKC1* mutations and also indicated that a genotoxic stress such as that induced by MMC amplified the phenotypic effects of these mutations. Additionally, the modeled phenotype reflects a functionally imbalanced progenitor state, rather than a complete loss of clonogenic capacity.

Next, we investigated the telomere length in individual colonies generated after *DKC1* editing. Compared with the HD group, both *DKC1* models generated colonies with shorter telomeres, which in some cases reached statistical significance (Figure 1D).

In subsequent studies, *DKC1*-edited cells were maintained in liquid cultures for up to 30 days post-nucleofection (Figure 2). When samples were edited using the NHEJ approach, a progressive decrease in the proportion of cells with *DKC1* indels was observed (Figure 2A). Similar decreases of cells with *DKC1* indels were observed when samples were edited according to the HDR model, which were compensated not only by increases of WT *DKC1* cells, but also by cells with the missense mutation (Figure 2B), showing the partial functionality of the hypomorphic dyskerin.

Analysis of the cell growth of these liquid cultures showed that both X-DC-like models exhibited significantly reduced expansion compared with mock-transduced CD34$^+$ cells (Figure 2C). The smaller reduction seen in the NHEJ condition is consistent with the lower fraction of *DKC1*-mutant cells in this model, relative to the HDR one (see A and B in Figure 2).

Since dyskerin plays a key role in ribosomal RNA synthesis, in particular in rRNA pseudouridylation and maturation, we also assessed the impact of *DKC1* mutations on ribosomal RNA maturation in edited cells.[34–36] To assess four pre-rRNA maturation fractions, PCR amplicons were designed. These amplicons enabled quantification of distinct pre-rRNA species based on probe specificity and differences in molecular weight, as resolved by northern blot (see Table S1).[37–39] As a control, we evaluated the accumulation of ribosomal RNA maturation fractions in BM cells from a *RPS19*-mutated DBA patient (a ribosomopathy characterized by defective 18 S rRNA maturation)[34,40,41] (See Figure S1C). Quantitative PCR (qPCR) analyses performed after 20 days of incubation showed the accumulation of intermediate ribosomal RNA maturation fractions in both

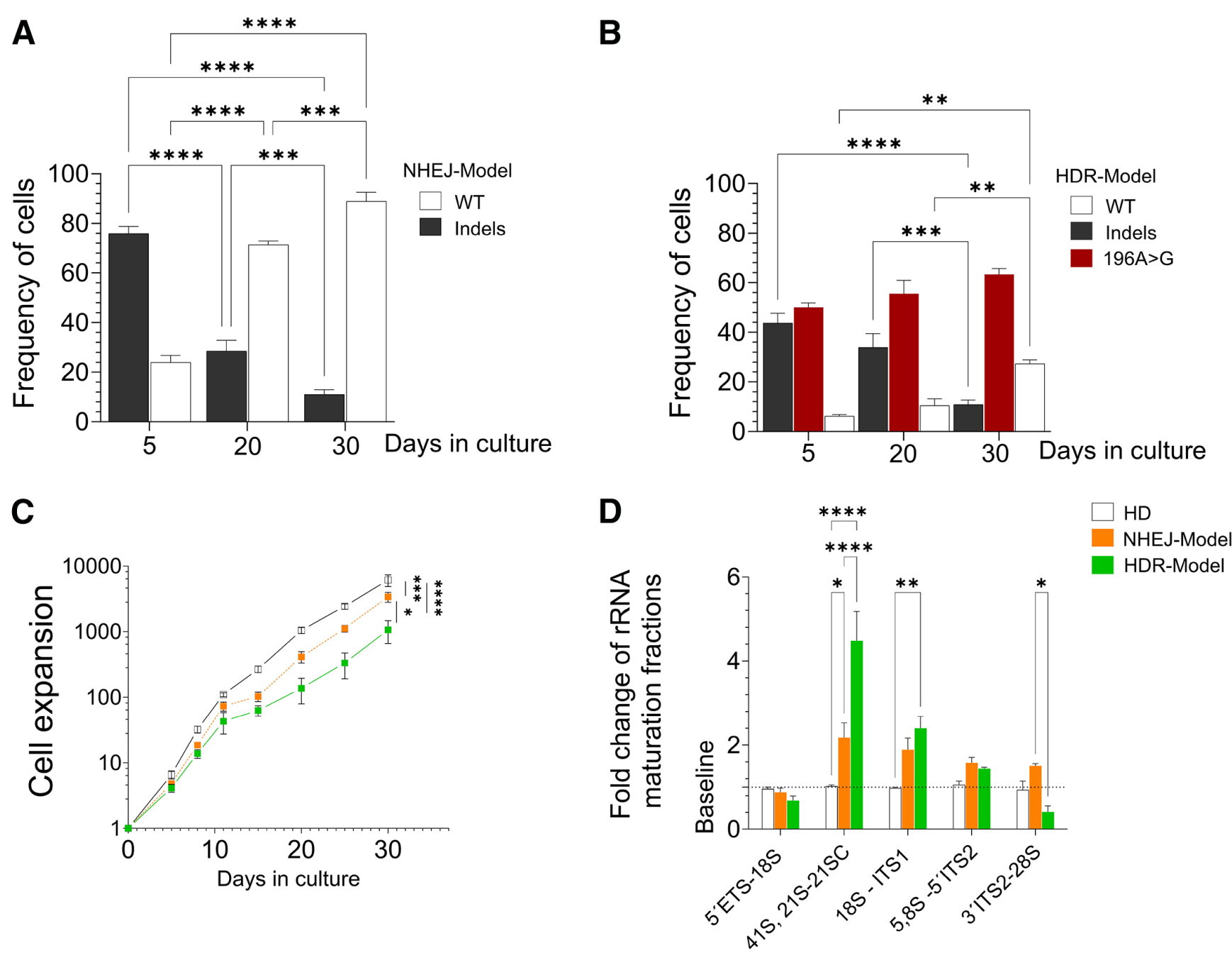

Figure 2. *In vitro* expansion defects and impaired ribosomal RNA maturation in *DKC1*-mutated CD34$^+$ cells

(A) Dynamics of *DKC1* genotype representation during liquid culture of NHEJ-edited CD34$^+$ cells. The frequency of WT and indel-bearing cells was determined at the indicated time points by Sanger deconvolution/qPCR. Data represent mean ± SEM from independent experiments. (B) Dynamics of *DKC1* genotype representation during liquid culture of HDR-edited CD34$^+$ cells. The frequency of WT, indel-bearing, and c.196 A>G cells was determined at the indicated time points by Sanger deconvolution/qPCR. Data represent mean ± SEM from independent experiments. (C) Expansion of non-edited HD CD34$^+$ cells and *DKC1*-mutated CD34$^+$ cells generated in the NHEJ and HDR models during standard liquid culture. Data represent mean ± SEM from four independent experiments. Statistical analysis was performed using two-way ANOVA with Tukey's post-hoc test. (D) Ribosomal RNA maturation defects in *DKC1*-mutated cells after 20 days in liquid culture, assessed by RT-qPCR analysis of pre-rRNA processing intermediates. Data are shown relative to HD controls. Mean ± SEM from 4 independent experiments is shown. Statistical analysis was performed using two-way ANOVA with Fisher's LSD post-hoc test. Detailed assay design is provided in materials and methods, Figure S1C, and Table S1.

DKC1-editing models. Although a higher accumulation of rRNA intermediates was observed in the HDR-model (Figure 2D), this likely reflects the higher proportion of DKC1-mutant cells in this group compared to NHEJ-treated cells. Given that both indel and hypomorphic mutations are generated in the HDR model, the contribution of each mutation type to the ribosomal RNA maturation defects observed in X-DC-like HSPCs is currently unknown.

Taken together, the results obtained in these studies indicate that the proposed gene-editing approaches constitute an efficient tool to generate X-DC-like HSPCs harboring *DKC1* indel and *DKC1* missense mutations previously identified in X-DC patients. In addition, our *in vitro* data demonstrate that compared with the c.196 A>G *DKC1* missense mutation, *DKC1* indels result in more pronounced phenotypic defects in human HSPCs.

The ectopic dyskerin expression mediated by the *TERC* promoter corrects the molecular and cellular phenotypes of X-DC-like HSPCs

To evaluate whether ectopic dyskerin expression corrects the functional defects of X-DC-like HSPCs, we generated therapeutic lentiviral vectors (LVs) carrying the WT version of the human *DKC1* cDNA. Because most X-DC mutations identified in patients are hypomorphic and may produce dyskerin variants with residual functionality, we aimed to increase wild-type dyskerin expression to levels sufficient to achieve functional complementation in the context of endogenous mutant protein expression. To this end, the therapeutic *DKC1* cDNA was placed under the control of two distinct heterologous promoters. Given the ubiquitous expression of dyskerin, we first selected the phosphoglycerate kinase (*PGK*) promoter, a widely used constitutive promoter that has been successfully used in previous clinical GT trials.[42] In addition, because *TERC* is constitutively expressed in human cells and encodes an essential component of the telomerase complex, a 798-bp fragment of the *TERC* promoter (pTERC) was also employed. As controls, EGFP-expressing LVs driven by the same promoters were generated (see schematic representation of the vectors in Figure S2A). Analysis of endogenous expression of telomerase-related genes in healthy cord-blood CD34$^+$ cells showed that *TERC* is expressed at higher levels than *DKC1* and other telomere-maintenance genes, supporting the rationale for using the *TERC* promoter to drive therapeutically relevant dyskerin expression in X-DC cells (Figure S2B).

To compare the transcriptional strength of the *TERC* promoter relative to the PGK promoter, we first evaluated EGFP expression in healthy CD34$^+$ cells previously transduced with pPGK-EGFP or pTERC-EGFP LVs. As shown in Figure S2C, the *PGK* promoter mediated substantially higher EGFP expression levels than the *TERC* promoter. In parallel experiments, CD34$^+$ cells were transduced with pPGK-DKC1 or pTERC-DKC1 vectors. Both vectors increased dyskerin mRNA levels above the baseline observed in control (pPGK-EGFP transduced) cells, with the pPGK-DKC1 vector driving higher transcript levels (Figure S2D). Consistent with these findings, western blot analyses in transduced murine 3T3 cells showed that the pPGK-DKC1 vector yielded higher dyskerin protein levels than the pTERC-DKC1 vector (Figure S2E).

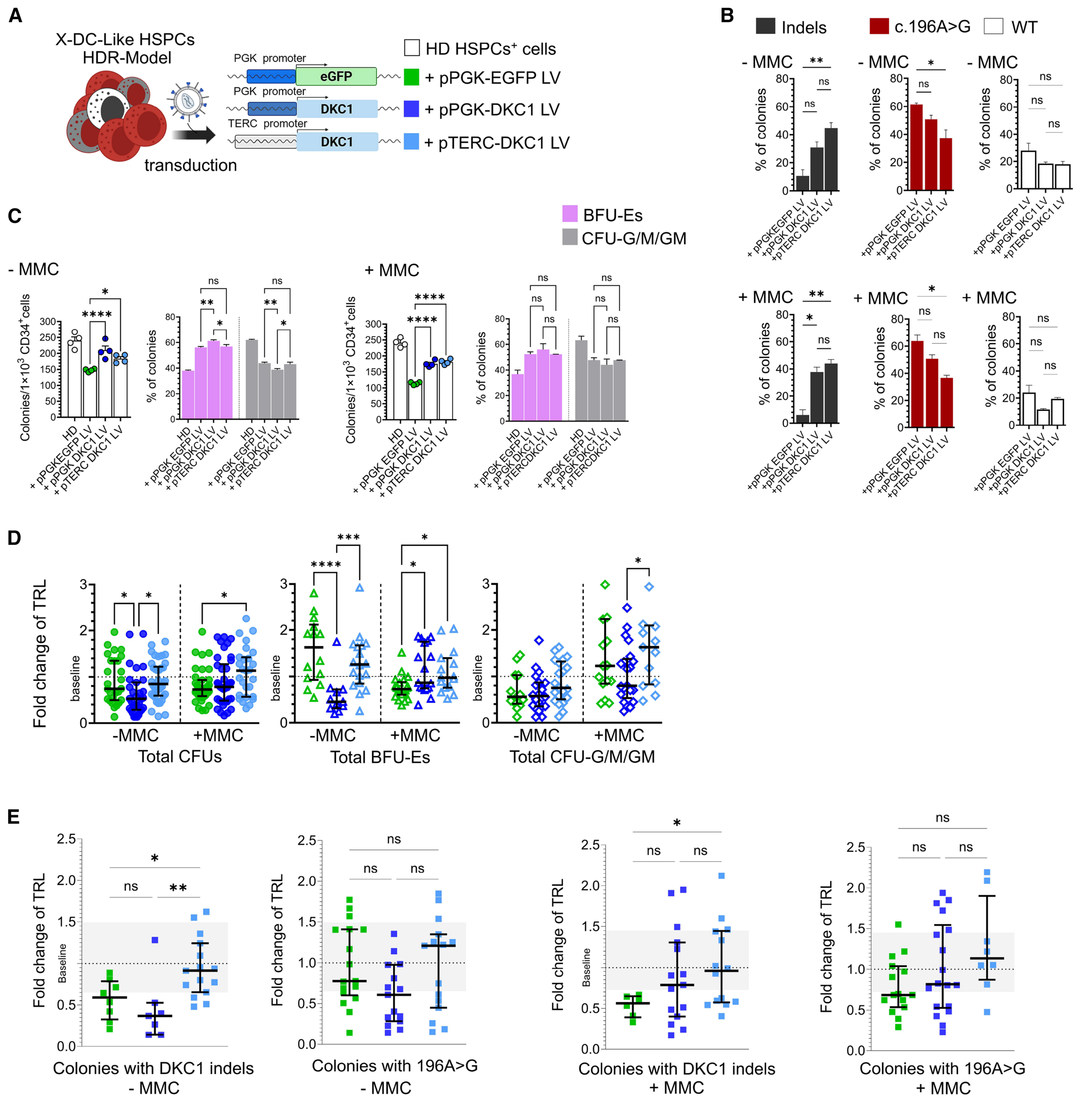

Figure 3. Phenotypic correction of X-DC-like hematopoietic colony-forming cells mediated by *DKC1* lentiviral vectors

(A) Experimental design. HDR-edited X-DC-like CD34+ cells were transduced with a control lentiviral vector-expressing EGFP (pPGK-EGFP LV) or therapeutic vectors expressing *DKC1* under the PGK promoter (pPGK-DKC1 LV) or the TERC promoter (pTERC-DKC1 LV). Unedited/untransduced HD CD34+ cells were included as reference controls. After transduction, cells were plated in methylcellulose in the absence or presence of MMC. (B) Distribution of *DKC1* genotypes in hematopoietic colonies generated from the experimental groups shown in panel A, analyzed separately in the absence and presence of MMC (10 nM). Colonies were classified as indel bearing, c.196 A>G, or WT. Individual colonies were genotyped by qPCR. Data represent mean ± SEM from four independent experiments. Tukey's multiple-comparison correction was applied for statistical analysis. (C) Clonogenic output of HD and X-DC-like CD34+ cells transduced with the indicated lentiviral vectors in the absence or presence of MMC. Left part of the image shows total colony numbers. Right part of the image shows the relative distribution of BFU-E and CFU-G/M/GM colonies within each condition. Data represent mean ± SEM from four independent experiments. (D) Telomere length in total colonies, BFU-E colonies, and CFU-G/M/GM colonies generated in the absence or presence of MMC,

(legend continued on next page)

Building on these promoter-comparison studies, we next evaluated the ability of the pPGK-DKC1 and pTERC-DKC1 LVs to correct the phenotype of X-DC-like HSPCs. In all these experiments, CB CD34$^+$ cells were edited using the HDR-based strategy shown in Figure 1A and were subsequently transduced with either therapeutic vector or with the pPGK-EGFP control vector (Figure 3A). The HDR-model was selected for subsequent experiments as it introduces a clinically relevant hypomorphic mutation and generates a heterogeneous allelic configuration, enabling the simultaneous assessment of therapeutic effects across both hypomorphic and more deleterious *DKC1* editing outcomes. Clonogenic assays showed transduction efficiencies ranging from 60% to 85% across all LVs (Figure S3A), resulting in mean vector copy numbers (VCN) of 1.4–2.0 copies per cell (Figure S3B). We then quantified, within each transduced group, the proportion of colonies carrying *DKC1* indel or missense mutations. As shown in Figure 3B, approximately 10% of colonies generated from EGFP-transduced control cells harbored *DKC1* indels. In contrast, the frequency of colonies carrying *DKC1* indels was increased in cells transduced with therapeutic DKC1 vectors, with the highest proportion observed in the pTERC-DKC1 group.

Conversely, colonies with either the *DKC1* missense mutation or the wild-type allele were more frequent in the EGFP-transduced control group compared with both therapeutic LV groups. A similar distribution was observed under MMC treatment, further supporting the ability of both therapeutic vectors to promote the growth of colonies harboring *DKC1* mutations (Figure 3B). Analysis of total colony numbers in both untreated and MMC-treated conditions showed increased colony formation in samples transduced with either therapeutic vector compared with the EGFP control LV (Figure 3C). Notably, in the therapeutic setting, lentiviral delivery increased total clonogenic output but did not reverse the altered colony composition of HDR-based X-DC-like HSPCs. Consistent with data obtained in the clonogenic assays, these findings indicate that *DKC1* indels impose a marked proliferative disadvantage and that the ectopic dyskerin expression mediated by either therapeutic LV overcomes this defect. However, the persistence of the relative erythroid enrichment suggests that, while therapeutic gene transfer restores proliferative capacity, it does not fully re-establish the intrinsic balance of X-DC-like progenitor output in the short term.

To determine whether the observed improvement in progenitor output was associated with changes in telomere maintenance, we next analyzed telomere length in individual hematopoietic colonies without prior stratification by genotype. Telomere length measurements across total colonies, as well as when stratified by colony type (BFU-E and CFU-G/M/GM), revealed that colonies derived from pTERC-DKC1-transduced cells displayed longer telomeres compared to those transduced with the EGFP control, whereas no consistent improvement was observed in colonies transduced with the pPGK-DKC1 vector (Figure 3D). This effect was observed across colony types, indicating that telomere preservation occurs at the level of clonogenic progenitors independently of lineage classification. To directly link these observations to the genetic status of individual colonies, we then analyzed telomere length in colonies with confirmed VCN and *DKC1* genotypes by Sanger sequencing. When colonies carrying *DKC1* indels were considered, we observed that the telomere length of cells harboring the pTERC-DKC1 provirus was significantly longer than that of cells harboring the EGFP provirus; a finding that was not observed in cells harboring the pPGK-DKC1 provirus (Figure 3E). Notably, the telomere length of pTERC-DKC1-transduced colonies was comparable to that of untreated HD-derived colonies. Analysis of colonies harboring the hypomorphic mutation revealed a similar trend, although differences did not reach statistical significance (Figure 3E). Similar trends were also observed in colonies generated under MMC treatment (Figure 3E). Taken together, these *in vitro* telomere length analyses indicate that the ectopic *DKC1* expression driven by the *TERC* promoter, but not by the *PGK* promoter, mitigates telomere attrition in X-DC-like HSPCs.

In addition to clonogenic assays, liquid cultures were established using X-DC-like CD34$^+$ cells transduced with either the EGFP or *DKC1* LVs. Droplet digital PCR (ddPCR) analysis revealed transduction efficiencies ranging from 70.9% to 78.6% (Figure S3C), resulting in VCN of 1.63–2.49 copies per cell (Figure S3D). In these cultures, we first assessed the expression of *DKC1*, *TERT*, and *TERC* five days after transduction. Compared with HD cells, EGFP-transduced X-DC-like cells displayed a mild increase in DKC1 mRNA levels together with a marked upregulation of *TERT* and, to a lesser extent, *TERC* expression, consistent with a compensatory activation of telomerase-related genes in response to dyskerin dysfunction (Figure S4A). As expected, dyskerin expression increased following transduction with either therapeutic vector, reaching the highest levels in cells transduced with the pPGK-DKC1 vector (Figure S4A). Notably, transduction with the pTERC-DKC1 vector, but not with the pPGK-DKC1 vector, reduced the elevated *TERT* and *TERC* RNA levels to values comparable to those observed in HD cells (Figure S4A). Consistent with these transcriptional changes, the *in vitro* telomerase activity was markedly increased in X-DC-like cells transduced with the EGFP control vector. This increase was normalized to near-healthy donor levels following transduction with the pTERC-DKC1 vector but not with the pPGK-DKC1 vector (Figure S4B).

Analysis of liquid cultures at 20 days post-transduction revealed a significantly higher proportion of cells carrying indels in samples that had been transduced with either therapeutic lentiviral vector

expressed as fold change relative to baseline. Each symbol represents an individual colony. Only colonies meeting telomere qPCR quality criteria were included (see materials and methods). Telomere length was measured by qPCR and expressed relative to HD baseline. Data correspond to individual colonies; horizontal bars indicate HD mean ± SEM. (E) Telomere length in individually genotyped colonies (Sanger deconvolution) carrying *DKC1* indels or the c.196 A>G hypomorphic mutation, analyzed separately in the absence and presence of MMC.

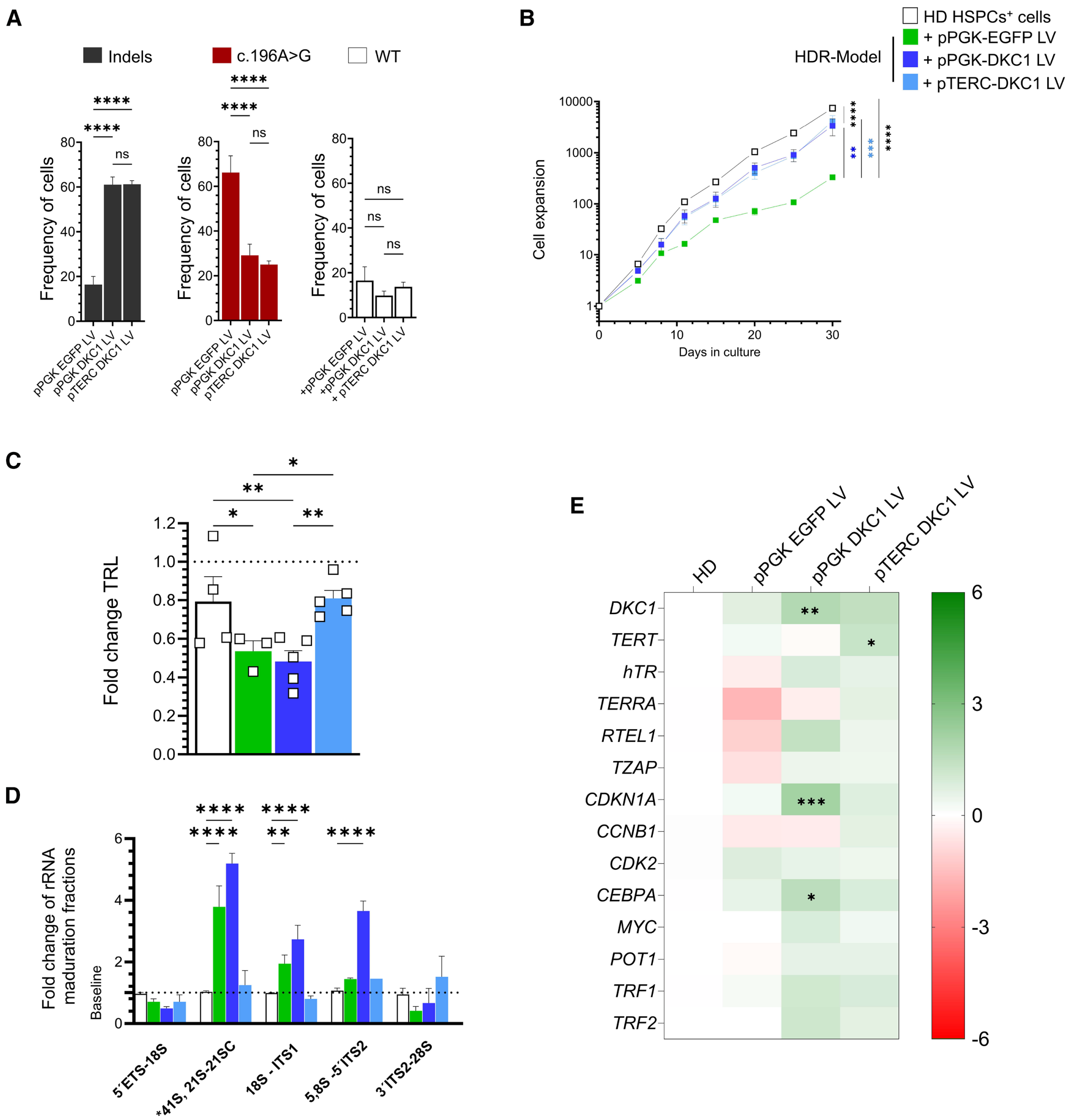

Figure 4. Phenotypic and molecular responses of X-DC-like CD34+ cells following transduction with *DKC1* lentiviral vectors and maintained in liquid cultures

(A) Frequency of indel-bearing, c.196 A>G, and WT cells in HDR-edited X-DC-like cultures 20 days after transduction with pPGK-EGFP LV, pPGK-DKC1 LV, or pTERC-DKC1 LV. Frequencies were determined in bulk cultures by Sanger deconvolution/qPCR. Data represent mean ± SEM. (B) Expansion of HD CD34+ cells and transduced X-DC-like CD34+ cells during 20 days of standard liquid culture. Data represent mean ± SEM from 4 independent experiments. (C) Telomere length at day 20 measured by qPCR and expressed as fold change relative to the starting CD34+ population before editing/transduction. Data are shown as mean ± SEM. (D) Pre-rRNA maturation profile at day 20 assessed by RT-qPCR in HD cells and in transduced HDR-model cells. Data show the relative abundance of the indicated pre-rRNA intermediates normalized to HD controls. (E) Heatmap showing RT-qPCR relative expression changes in genes related to telomere biology, cell-cycle regulation, and hematopoietic function in day-20 cultures. Expression values are shown as log2 fold change relative to HD cells. Statistical significance was assessed using two-way ANOVA followed by Dunnett's multiple-comparison correction. Asterisks indicate significance levels (*$p < 0.05$; **$p < 0.01$; ***$p < 0.001$).

compared with EGFP-transduced controls (Figure 4A), in line with the results of the clonogenic assays shown in Figure 3. In parallel, the impaired growth kinetics observed in EGFP-transduced X-DC-like HSPCs were improved following transduction with either therapeutic vector (Figure 4B). The telomere length was also evaluated at this time point in corrected and uncorrected X-DC-like cells. EGFP-transduced cells displayed marked telomere attrition relative to HD controls. This telomeric shortening was, however, not observed in cells transduced with the pTERC-DKC1 vector, whereas the pPGK-DKC1 vector did not provide comparable protection (Figure 4C).

Using the same experimental conditions, rRNA maturation was next examined in these samples. EGFP-transduced X-DC-like cells showed an accumulation of rRNA maturation intermediates, consistent with the alterations observed in experiments of X-DC modeling (Figure 2D). This accumulation was resolved in cells transduced with the pTERC-DKC1 vector, whereas the pPGK-DKC1 vector failed to produce a comparable effect (Figure 4D). To further examine the differential effects of the pPGK-DKC1 and pTERC-DKC1 LVs on telomere maintenance and rRNA maturation defects in X-DC-like hematopoietic cells, we analyzed the expression of a selected panel of telomere- and cell-cycle-related genes by targeted RT-qPCR at 20 days post-transduction (Figure 4E). EGFP-transduced X-DC-like cells displayed a transcriptional profile consistent with telomere stress, characterized by reduced *TERC* and *TERRA* expression, in line with dyskerin-associated instability and impaired subtelomeric transcription previously described in TBDs.[43–46] In addition, these cells showed modest upregulation of *TERT*, *DKC1*, *RTEL1*, *TZAP*, and shelterin components, together with increased *CDK2* expression, reduced *CCNB1* levels, and elevated *CEBPA* expression, suggestive of altered cell-cycle regulation and early myeloid differentiation bias. Cells transduced with the pPGK-DKC1 vector, which drove higher levels of dyskerin expression, largely retained a stress-associated transcriptional profile. This included persistent *TERRA* repression, sustained *RTEL1* upregulation, further elevation of *CEBPA*, evident induction of *CDKN1A*, moderate *MYC* upregulation, and continued downregulation of *CCNB1* despite preserved *CDK2* expression. By comparison, pTERC-DKC1 transduction was associated with a transcriptional profile lacking the coordinated stress-associated changes observed in pPGK-DKC1-transduced samples.

These transcriptional differences are consistent with the distinct functional outcomes observed for the two therapeutic vectors in assays of telomere length and rRNA maturation (Figures 3 and 4; Figure S4A). Overall, whereas both therapeutic LVs enhanced the proliferative capacity of X-DC-like HSPCs, only the pTERC-DKC1 vector was associated with preservation of telomere length and correction of the rRNA-maturation defects characteristic of X-DC-like cells.

Sustained correction of X-DC-like HSPCs following transduction with the pTERC-DKC1 lentiviral vector

Given that DC HSPCs are characterized by impaired self-renewal, serial replating progenitor assay serves as an *in vitro* functional readout of progenitor fitness prior to and after gene correction. To assess therapeutic effects in more primitive X-DC-like progenitors, serial replating assays were performed (Figure 5A). As expected in these replating assays, the total number of secondary colonies was markedly lower than in primary cultures, and did not show significant differences between experimental conditions (Figure 5B). Although not statistically significant, uncorrected X-DC-like cells showed a consistent trend toward increased erythroid output upon replating, in line with primary clonogenic assays (Figures 1C and 3C), which was normalized following pTERC-DKC1 transduction upon serial replating. Notably, secondary colonies derived from pTERC-DKC1-transduced cells exhibited a significant increase in the VCN per cell relative to their corresponding primary colonies, an effect not observed in the EGFP-transduced group (Figure 5C). As VCN was determined in pooled colonies, these values reflect average proviral copy number across the total progenitor population rather than individual progenitor cells. This enrichment suggests a selective proliferative advantage of X-DC-like progenitors harboring the pTERC-DKC1 provirus. Analysis of *DKC1* mutations in primary and secondary colonies derived from EGFP-transduced X-DC-like $CD34^+$ cells revealed a marked reduction in the proportion of colonies carrying *DKC1* indels and, to a lesser extent, of those harboring the hypomorphic mutation upon replating (Figure 5D). In contrast, this reduction was not observed in pTERC-DKC1-transduced samples, indicating that pTERC-driven dyskerin expression supported the replating capacity of hematopoietic progenitors carrying either type of *DKC1* mutation. Telomere length analysis in primary and secondary colonies showed that those derived from the pTERC-DKC1 group were significantly longer relative to EGFP-transduced controls (Figure 5E), further supporting the functional rescue of X-DC-like HSPCs mediated by this therapeutic vector.

To evaluate the *in vivo* performance of X-DC-like HSPCs and assess the therapeutic effects of pTERC-DKC1 LV transduction, we followed the experimental strategy outlined in Figure 6A. Healthy-donor and X-DC-like $CD34^+$ cells transduced with a control non-therapeutic LV were included as reference groups (see materials and methods for full details). As shown in Figures 6B and 6C, engraftment of X-DC-like HSPCs (% of human $CD45^+$ and $CD34^+$ cells) in recipient BM was markedly lower than in the HD group, irrespective of whether cells were transduced with the control or the therapeutic LV. This reduction most likely reflects the extensive $CD34^+$-cell manipulation required for the introduction and subsequent correction of *DKC1* mutations in this experimental approach. Analysis of lineage distribution within the engrafted $hCD45^+$ compartment revealed a skewed hematopoietic output in X-DC-like grafts, characterized by reduced $hCD19^+$ cells and relatively increased myeloid and T cell contributions at later time points. This imbalance was not overtly normalized by pTERC-DKC1 within the time frame of the *in vivo* assay, although corrected grafts showed higher $hCD33^+$ and $hCD3^+$ representation than uncorrected controls (Figures 6D–6F). Given the limited duration and engraftment of this model, longer-term effects on lineage balance may not be fully captured. Consistent with this, serial clonogenic assays (Figure 5B) indicate that dyskerin correction supports normalization of lineage output under extended proliferative stress.

Subsequent analyses therefore focused on determining whether pTERC-DKC1 transduction conferred a sustained functional correction within the engrafted X-DC-like hematopoietic compartment over time. In these studies, a progressive increase in the VCN per human cell was observed in BM-engrafted cells from recipients transplanted with pTERC-DKC1-transduced cells, an effect that was not detected in the control-LV group (Figure 7A). Analysis of *DKC1* genotypes showed that cells carrying indels were completely lost over time in the control-LV group, whereas these mutant alleles persisted

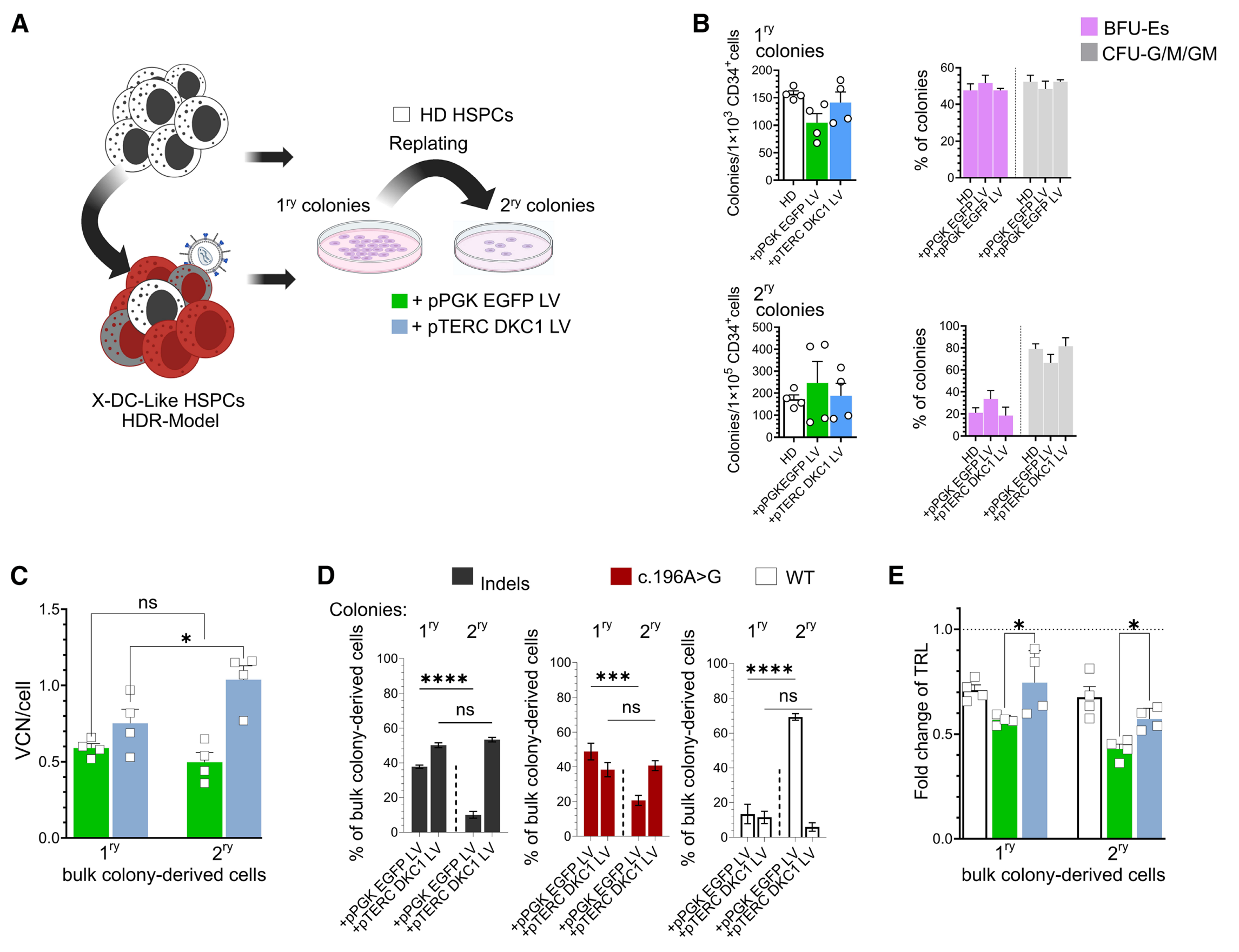

Figure 5. Serial replating reveals persistence of gene-corrected X-DC-like progenitors *in vitro*

(A) Schematic representation of the serial replating assay. HDR-edited X-DC-like CD34+ cells were transduced with pPGK-EGFP LV or pTERC-DKC1 LV and plated in primary methylcellulose cultures. Cells recovered from primary colonies were replated into secondary methylcellulose cultures. Mock-electroporated HD CD34+ cells exposed to the donor template but not edited were included as reference controls. (B) Number of primary and secondary colonies generated in each condition. Right part of the image shows the relative distribution of BFU-E and CFU-G/M/GM colonies in primary and secondary clonogenic cultures. Data represent mean ± SEM from 4 independent experiments. (C) Lentiviral vector copy number per cell (VCN/cell) measured in bulk of cells recovered from primary and secondary colony assays. Data represent mean ± SEM. Statistical comparisons among groups were performed using one-way ANOVA followed by Bonferroni's post hoc test. (D) Distribution of *DKC1* genotypes in cells recovered from primary and secondary colony assays. Genotype frequencies were determined by Sanger deconvolution/qPCR. Data represent mean ± SD from pooled colonies. Statistical comparisons were performed using two-way ANOVA with Tukey's multiple-comparison correction. (E) Telomere length in cells recovered from primary and secondary colony assays, measured by qPCR and expressed relative to baseline. Data are shown as mean ± SEM. Statistical analysis was performed using Fisher's least significant difference (LSD) multiple-comparison test.

within the engrafted cells receiving the therapeutic vector (Figure 7B). Cells harboring the hypomorphic mutation were also markedly reduced in the control LV group, revealing the competitive repopulation disadvantage of these cells. In contrast, in the pTERC-DKC1-transduced group, the proportion of cells carrying *DKC1* indel or hypomorphic mutations was maintained throughout the four-month follow-up period (Figure 7B). These results reveal the impaired engraftment capacity of HSPCs harboring *DKC1* mutations, particularly of frameshift indels, and demonstrate that pTERC-DKC1 transduction supports the repopulation of HSPCs harboring *DKC1* mutations.

In these xenotransplantation experiments, telomere length was also evaluated in engrafted human hematopoietic cells using the flow-FISH approach described in materials and methods (Figure S5). In the HD group consisting of non-*DKC1*-edited cells, the telomere length progressively declined to approximately 60% of pre-transplant values at four months post-transplantation in both hCD45+ and hCD34+ compartments, with faster attrition observed in hCD45+ cells (Figures 7C and 7D). In the X-DC-like groups, the telomere length of engrafted hCD45+ cells was markedly shorter than that of non-edited controls, and no differences were detected between cells transduced with the control or therapeutic vectors (Figure 7C). By contrast, in the hCD34+ compartment, X-DC-like progenitors transduced with the pTERC-DKC1 LV displayed significantly longer telomeres compared with the corresponding control LV-group, and at four months post-transplantation were comparable to those

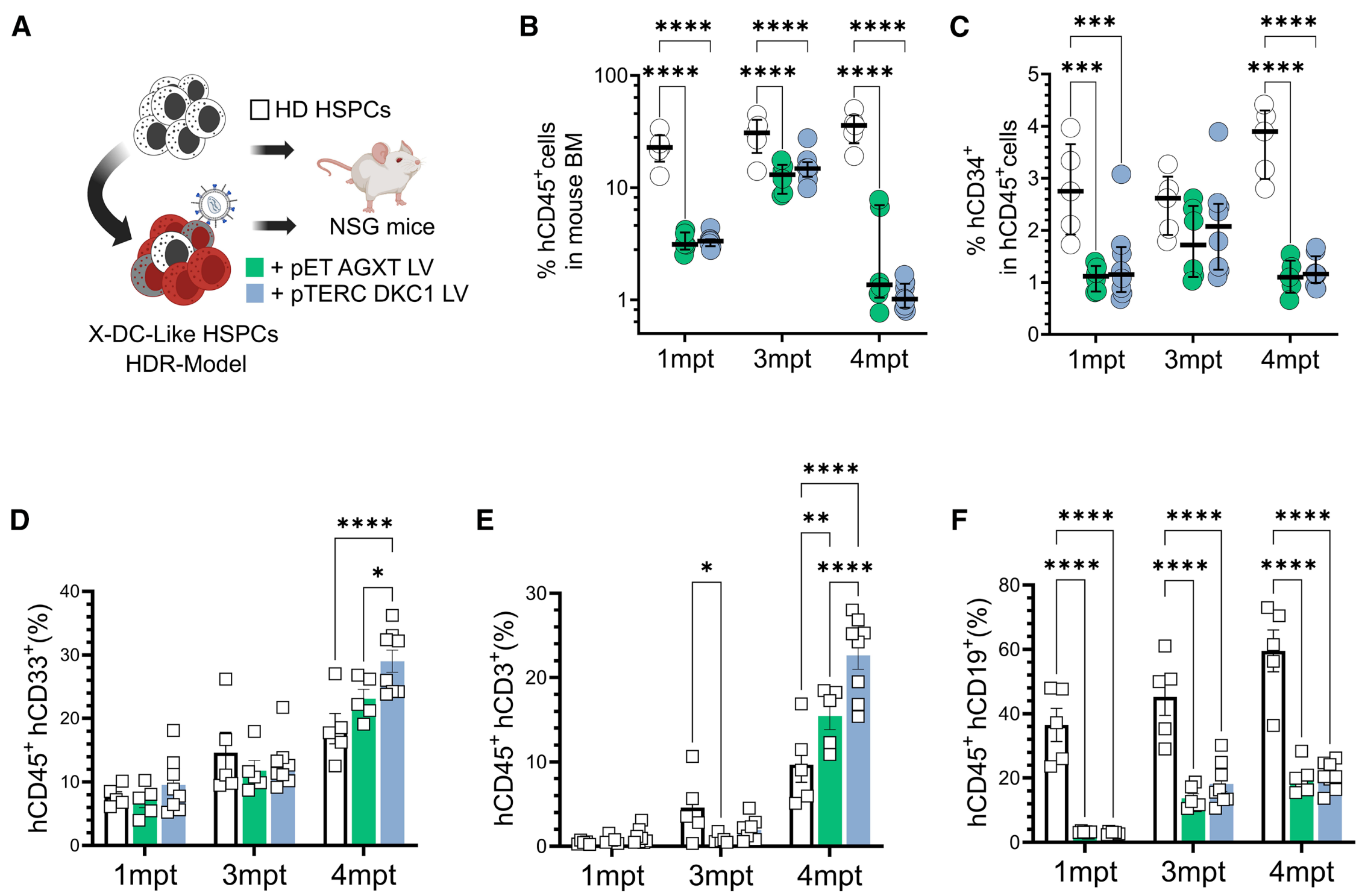

Figure 6. *In vivo* engraftment and lineage output of X-DC-like HSPCs following lentiviral correction

(A) Experimental design used to evaluate the *in vivo* effects associated with transduction X-DC-like CD34$^+$ cells with the pTERC-DKC1 LV. X-DC-like CD34$^+$ cells were transduced with either a control LV (pET-AGXT; see materials and methods) or the therapeutic pTERC-DKC1 LV and transplanted into sublethally irradiated NSG mice (control LV group, n = 6 mice; pTERC-DKC1 group, n = 8 mice). As an additional control, mock-electroporated CD34$^+$ cells (including exposure to the mT donor used for X-DC modeling) and subsequently transduced with the non-therapeutic pET-AGXT LV (MOI = 20) were transplanted into five mice. In all groups, 5 × 10^5 cells were infused per mouse. (B) Human hematopoietic engraftment in bone marrow, shown as the percentage of hCD45$^+$ cells in recipient mice at the indicated post-transplant time points. (C) Frequency of human CD34$^+$ cells within the engrafted hCD45$^+$ compartment at the indicated post-transplant time points. (D) Myeloid lineage contribution within engrafted human cells, assessed as the percentage of hCD33$^+$ cells in the hCD45$^+$ compartment. (E) T cell lineage contribution within engrafted human cells, assessed as the percentage of hCD3$^+$ cells in the hCD45$^+$ compartment. (F) B cell lineage contribution within engrafted human cells, assessed as the percentage of hCD19$^+$ cells in the hCD45$^+$ compartment. Data represent mean ± SEM. Statistical comparisons among groups were performed using two-way ANOVA followed by Tukey's post-hoc test.

determined in unmanipulated control samples (Figure 7D). These findings are consistent with the skewed lineage composition of X-DC-like grafts (Figures 6D–6F), where increased myeloid and T cell output, reflecting higher proliferative turnover, results in a greater cumulative replicative history in the bulk hCD45$^+$ compartment of pTERC-DKC1-corrected cells relative to uncorrected grafts, thereby reducing the resolution of telomere differences at this level despite clear preservation in progenitor cells.

Taken together, the *in vitro* replating and *in vivo* transplantation studies demonstrate the proliferative and repopulating defects associated with *DKC1* mutations. These defects were more pronounced in cells harboring *DKC1* indels, although were also evident, albeit to a lesser extent, in cells carrying the hypomorphic c.196 A>G mutation. Notably, transduction with the pTERC-DKC1 LV preserved the telomere length to values similar to those characteristic of HD cells and enabled the repopulation of *DKC1*-mutated CD34$^+$ cells.

Transduction of healthy HSPCs with pTERC-DKC1 LV does not modify the phenotypic properties of these precursor cells

To investigate whether DKC1 overexpression causes adverse effects in human HSPCs, HD CD34$^+$ cells already expressing physiological dyskerin levels were transduced with the pTERC-DKC1 LV or with a control EGFP LV. Thereafter, primary and secondary clonogenic assays were conducted to evaluate the *in vitro* phenotypic properties of the corresponding progenitor cells (see experimental design in Figure 8A). While data in Figure S2D showed that the pTERC-DKC1 LV drives supraphysiological dyskerin levels in HD CD34$^+$ cells, no differences were observed in colony numbers, VCN/cell, or telomere length across primary and secondary colonies (Figures 8B–8D) between the pTERC-DKC1 and the pPGK-EGFP LV groups.

Additional *in vivo* studies were performed using bone marrow samples from NSG mice transplanted with HD CD34$^+$ cells transduced with either the control pET-AGXT or the pTERC-DKC1 LV. As shown in Figure 8E, VCN per human bone marrow cell

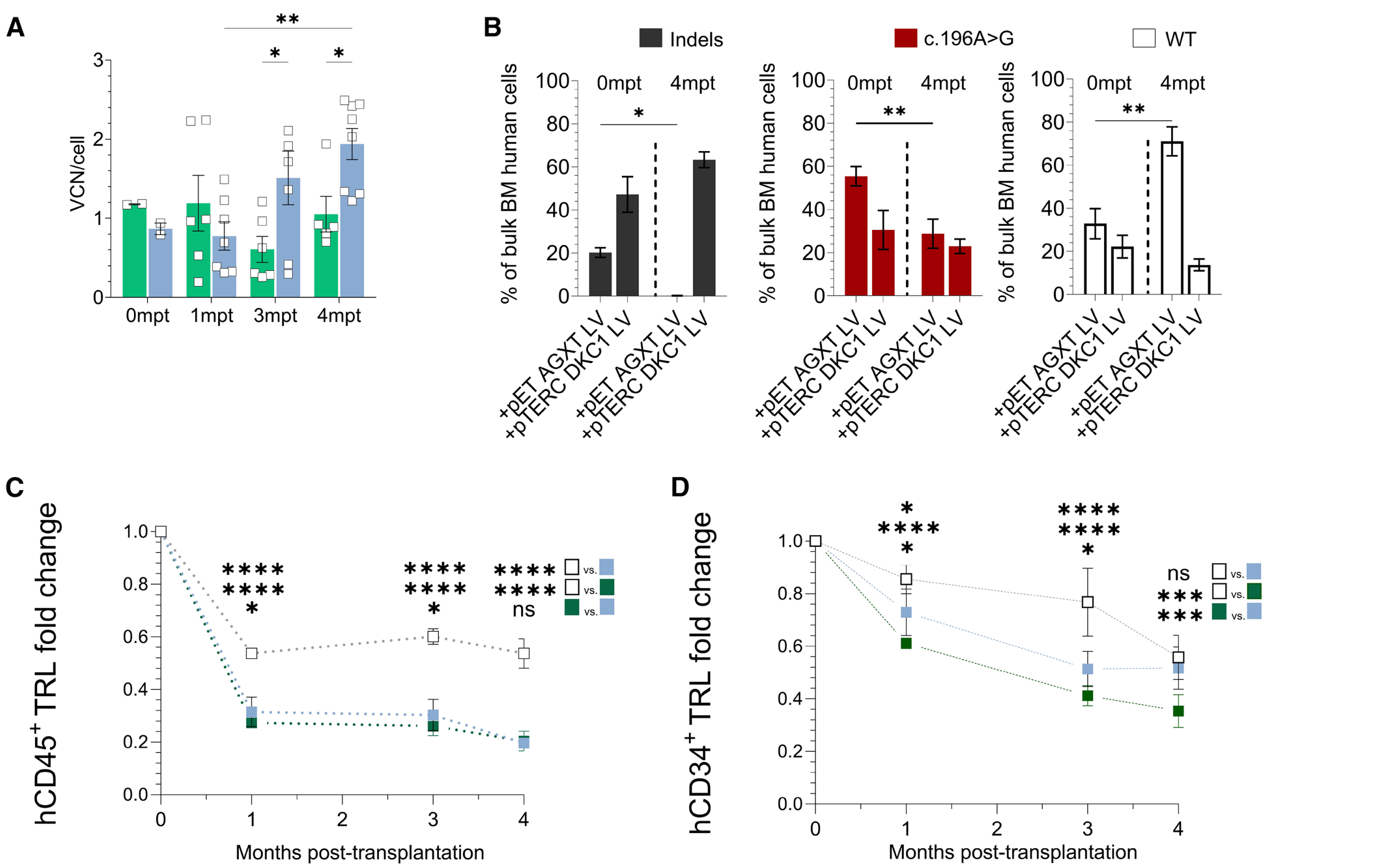

Figure 7. *In vivo* clonal dynamics and telomere length in engrafted X-DC-like HSPCs

(A) Vector copy number per human hematopoietic cell (VCN/cell) measured in BM samples from recipient mice transplanted with the indicated donor cell populations at sequential post-transplant time points. Tukey's multiple-comparison correction was applied to comparisons among LV-treated groups over time. (B) Distribution of *DKC1* genotypes in input cells before transplantation (0 mpt) and in engrafted human bone marrow cells at 4 months post-transplantation (4 mpt). Input samples were analyzed in edited cell preparations before transplantation, whereas 4-mpt analyzes were performed on bone marrow—derived cells recovered from recipient mice by Sanger deconvolution/qPCR. Data show mean ± SEM. Tukey's correction was used to assess changes in mutation burden over time within each treatment group. (C) Relative telomere length dynamics in engrafted human hCD45^{+} cells measured by Flow-FISH and expressed as fold change relative to input values. (D) Relative telomere length dynamics in engrafted human hCD45^{+}CD34^{+} cells measured by Flow-FISH and expressed as fold change relative to input values. The gating strategy used to discriminate human and mouse cells and to define hCD45^{+} and hCD45^{+}CD34^{+} compartments is shown in Figure S5. Plotted values represent mean ± SEM telomere length at sequential time points for transplanted mice. Fisher's least significant difference (LSD) test was used for overall comparisons, followed by appropriate post hoc analyses at individual time points.

remained comparable between groups throughout the 20-week follow-up period. Similarly, no differences were detected in the frequency of human CD45^{+} or CD34^{+} cells (Figures 8F and 8G), or in the distribution of differentiated hematopoietic lineages (Figure 8H). Analysis of telomere length in engrafted human cells revealed the expected progressive shortening over time in both cohorts, with no differences between groups (Figures 8I and 8J).

Collectively, these *in vitro* and *in vivo* data demonstrate that dyskerin overexpression mediated by the pTERC-DKC1 LV does not affect repopulation, differentiation, or telomere dynamics of healthy HSPCs that express physiological levels of dyskerin.

DISCUSSION

Advances in vector design and genome editing have made GT an increasingly important option for a range of monogenic disorders.[47] In the hematopoietic system, these developments have translated into clear clinical benefits for patients with primary immunodeficiencies, metabolic disorders, and hemoglobinopathies.[48,49] More recently, GT has been successfully extended to inherited BMF syndromes such as FA, in which gene-corrected multipotent HSCs achieve progressive engraftment by virtue of their proliferative advantage even in the absence of patient conditioning.[29,30,50] These observations thus provide a strong conceptual framework for GT of other BMF syndromes such as DC, where therapeutic benefit is likely to depend not only on gene correction itself but also on the baseline fitness and the availability of a sufficient residual pool of HSPCs capable of sustaining long-term hematopoiesis.

To establish a clinically relevant platform for GT evaluation in X-DC, we first evaluated the functional impact of *DKC1* mutations in human HSPCs. To this end, X-DC-like models were generated by introducing pathogenic *DKC1* variants into neonatal CD34^{+} cells, enabling the capture of early disease-associated defects in human

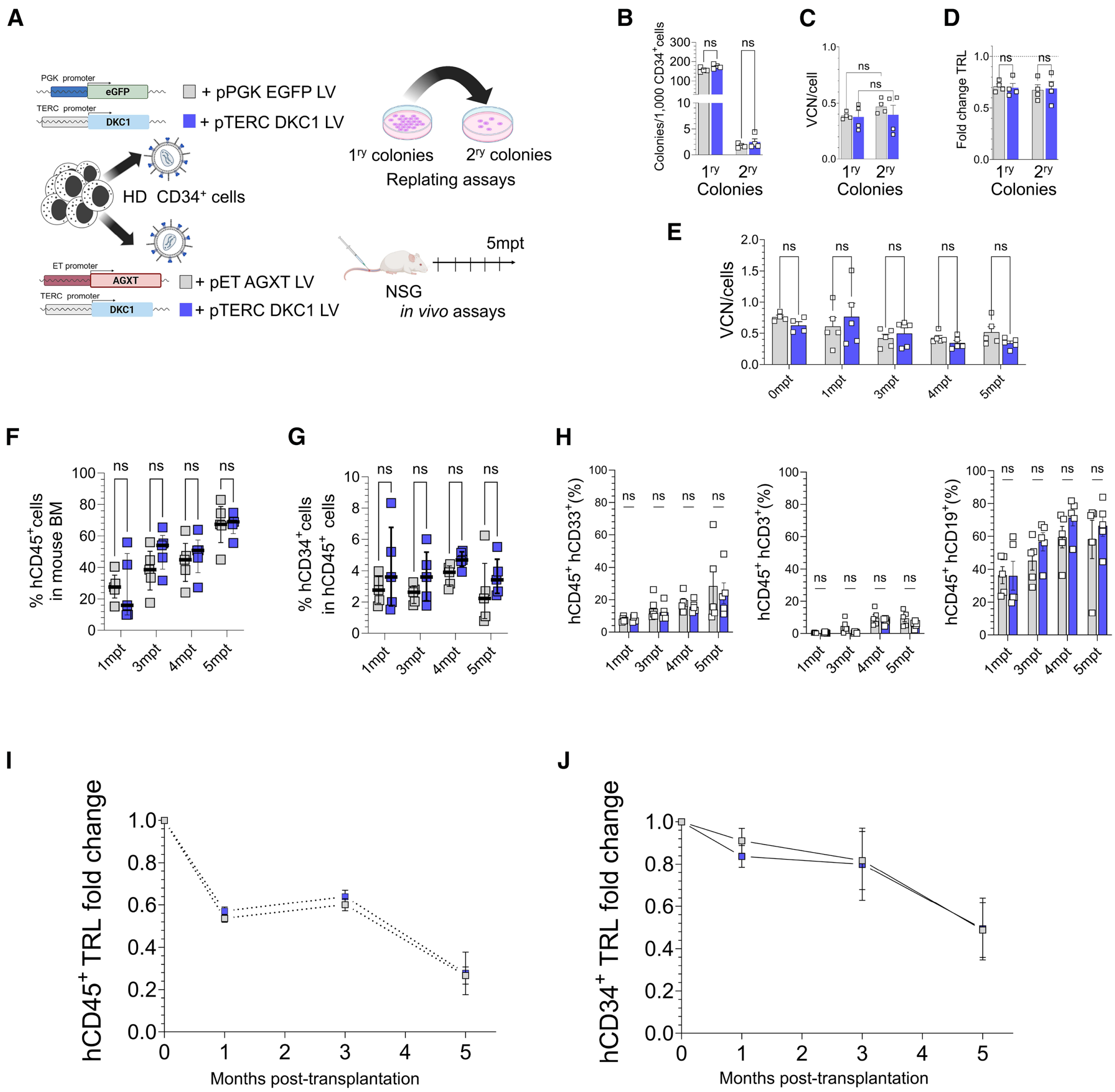

Figure 8. Functional assessment of healthy human HSPCs transduced with the pTERC-DKC1 lentiviral vector

(A) Experimental approach used to evaluate the effects of transduction of HD CD34$^+$ cells with the pTERC-DKC1 LV. Cord blood CD34$^+$ cells were mock-electroporated, including exposure to the mT donor used for X-DC modeling, and then transduced with either the control LVs (pPGK-EGFP for *in vitro* assays or pET-AGXT for *in vivo* assays) or the therapeutic pTERC-DKC1 LV. All transductions were performed at a MOI of 20. (B) Replating clonogenic capacity of HD CB CD34$^+$ cells following transduction with the control pPGK-EGFP LV or the therapeutic pTERC-DKC1 LV (n = 4 independent assays). (C) Lentiviral vector copy number per cell (VCN/cell) in cells recovered from primary and secondary colony assays. (D) Telomere length measured by qPCR in colonies corresponding to each experimental group in the serial replating assay. The horizontal dotted line indicates the normalized telomere length of the starting CD34$^+$ cell population prior to transduction. Bonferroni's correction was applied for multiple comparisons across experimental groups in (B)–(D). (E) VCN/Cell determined in BM of recipient mice transplanted with healthy CD34$^+$ cells transduced with pET AGXT LV or pTERC-DKC1 LV. (F) Human hematopoietic engraftment, shown as the percentage of hCD45$^+$ cells in bone marrow at the indicated time points. (G) Frequency of human CD34$^+$ cells within the engrafted hCD45$^+$ compartment. (H) Distribution of mature human hematopoietic lineages in bone marrow, including myeloid cells (hCD45$^+$CD33$^+$), T lymphocytes (hCD45$^+$CD3$^+$), and B lymphocytes (hCD45$^+$CD19$^+$), at the indicated post-transplant time points. Bonferroni's correction was applied to compare control and therapeutic groups at each time point. (I) Relative telomere length dynamics in engrafted hCD45$^+$ cells measured by flow-FISH. (J) Relative telomere length dynamics in engrafted hCD45$^+$CD34$^+$ cells measured by flow-FISH. LSD test was used for overall group comparisons independent of time.

HSPCs. This experimental setting provides a biologically relevant platform to directly compare distinct classes of *DKC1* mutations and to evaluate GT approaches at an early stage of disease, when telomere integrity is still preserved. While X-DC-like cells do not fully recapitulate the chronic telomere erosion and HSC exhaustion observed in patients, this platform enables the assessment of early therapeutic interventions, which are likely to be critical determinants of clinical efficacy in TBDs.

Clinically, XDC is predominantly associated with hypomorphic *DKC1* mutations that drive progressive HSPC attrition and, ultimately, BMF.[51–53] To interrogate the consequences of different degrees of dyskerin dysfunction, we modeled two classes of *DKC1* mutations: frameshift indels and the clinically reported hypomorphic Thr66Ala variant.[13,18,54] Importantly, the hypomorphic HDR-based model shown in this study generates a heterogeneous allelic landscape, allowing the simultaneous evaluation of disease hallmarks and therapeutic effects across the hypomorphic Thr66Ala variant and more deleterious frameshift outcomes within the same experimental setting. Indeed, introduction of frameshift mutations resulted in profound impairment of human HSPCs, manifested by severe proliferative defects *in vitro* and loss of repopulating capacity in immunodeficient NSG mice. In contrast, the Thr66Ala mutation caused more modest, though still significant reductions in HSPC proliferation and repopulation, closely recapitulating the disease course observed in patients carrying hypomorphic *DKC1* variants. Notably, consistent with the pleiotropic role of dyskerin in telomere maintenance and RNA biology,[6,35,55–57] *DKC1*-mutated HSPCs exhibited core hallmarks of X-DC, including impaired proliferation, accelerated telomere shortening, and defective rRNA maturation, all of which likely contribute to progressive BMF in patients with X-DC. In addition, *DKC1*-mutated cells displayed altered lineage output across experimental settings, with a relative enrichment of erythroid progenitors *in vitro* and a skewed differentiation pattern *in vivo* characterized by reduced B cell output and relative enrichment of myeloid and T cell compartments. Although not fully recapitulating patient hematopoiesis, these features are consistent with the erythro-megakaryocytic bias and lymphoid depletion described in telomere-dysfunctional mouse HSPCs at single-cell resolution,[58] and with lymphoid deficiencies reported in X-DC patients.[59]

Using the HDR-based model of X-DC-like HSPCs, we next compared the efficacy and safety of two LVs carrying the *DKC1* gene, which conferred different levels of dyskerin expression, pPGK-DKC1 and pTERC-DKC1, the latter driven by regulatory elements of the *TERC* locus, in the context of progressive X-DC-like phenotypes emerging under proliferative conditions. While transduction with either vector improved proliferative output of X-DC-like HSPCs *in vitro*, only *TERC* promoter-driven dyskerin expression efficiently returned telomerase activity and rRNA maturation toward near-physiological levels. In contrast, dyskerin expression driven by the stronger *PGK* promoter failed to fully correct these molecular hallmarks, despite improving short-term proliferation of *DKC1*-mutated HSPCs. These findings indicate that increased proliferative output alone does not necessarily reflect functional correction in X-DC HSPCs, and highlight the importance of achieving near-physiological dyskerin expression to restore both telomeric and extratelomeric functions. Notably, dyskerin expression driven by the *TERC* promoter supported stable molecular correction within the experimental setting without detectable perturbation of cellular homeostasis, highlighting a functionally tolerated expression window as a key determinant of therapeutic efficacy in X-DC. These findings support a model in which dyskerin expression must be maintained within a functionally tolerated range to ensure coordinated telomerase activity and RNP homeostasis, thereby defining a functional therapeutic window for GT in X-DC. Although the underlying mechanisms were not directly addressed in this study, these observations are compatible with the notion that excessive dyskerin expression may perturb RNP complex homeostasis, potentially through altered stoichiometry of H/ACA complexes or sequestration of limiting small nucleolar RNAs required for rRNA maturation and pseudouridylation.[6,57,60–62]

Previous attempts to provide dyskerin complementation using vectors with strong promoters or plasmid-based delivery systems have resulted in incomplete or inconsistent restoration of telomere-associated functions.[62,63] In contrast, the use of weaker promoters, such as those derived from the *TERC* locus, allowed us to achieve dyskerin expression levels that were compatible with efficient phenotypic correction in *DKC1*-mutated HSPCs, including rescue of telomere defects and improved RNP complex homeostasis. Importantly, this correction was not associated with detectable adverse effects in healthy $CD34^+$ cells, which maintain physiological dyskerin levels, further supporting the safety of this GT strategy.

The functional benefits conferred by pTERC-DKC1 extended beyond *in vitro* assays. In xenotransplantation experiments, despite the limited engraftment of extensively manipulated cells, transduction of X-DC-like HSPCs with the pTERC-DKC1 LV facilitated the repopulation of *DKC1*-mutated cells and preserved telomere integrity within the experimental time frame, consistent with a functional advantage of corrected cells in a competitive hematopoietic environment. Notably, corrected grafts displayed increased contribution of myeloid and T cell compartments compared with uncorrected X-DC-like cells, whereas the reduced B cell fraction was not restored within the assay window, reflecting limitations of the *in vivo* model to capture longer-term lineage rebalancing. Although sequential transplants could not be performed due to limited human engraftment, complementary *in vitro* assays, including serial replating and longitudinal VCN analysis, support the sustained functional advantage of corrected progenitors.

Since our preclinical studies demonstrated phenotypic correction in HSPCs carrying both the hypomorphic c.196 A>G (Thr66Ala) variant and the highly pathogenic *DKC1* indels, our findings indicate that the pTERC-DKC1 LV can effectively correct distinct classes of *DKC1* mutations in human HSPCs. Unlike telomere-elongation strategies such as ZSCAN4 activation,[64] the approach described here restores multiple dyskerin-dependent functions, enabling concurrent correction of telomerase activity and ribosome-associated defects characteristic of X-DC cells.

From a translational perspective, although CB-derived $CD34^+$ cells provide a controlled and homogeneous system to model early disease-associated defects, clinically relevant autologous GT approaches would most likely rely on mobilized peripheral blood or bone marrow-derived HSPCs.[29,64] Our findings are conceptually aligned

with GT studies in FA, in which corrected HSPCs progressively outcompete uncorrected counterparts without the need for pre-conditioning.[30,31,50] In X-DC, and likely in other telomere biology disorders, gene correction is expected to restore telomere maintenance while conferring a proliferative advantage to corrected HSPCs, thereby supporting their selective persistence and contribution to hematopoiesis over time. In this context, therapeutic efficacy is likely to depend strongly on the baseline quantity and functional reserve of the HSC compartment.

Taken together, the human X-DC models established in this study recapitulate the core biological features of X-DC patient-derived HSPCs and provide a robust platform for mechanistic and therapeutic evaluation. Our findings further show that dyskerin expression levels driven by TERC regulatory elements are compatible with the restoration of proliferative capacity, telomere maintenance, and rRNA maturation, thereby preserving HSPC homeostasis. Although additional validation studies using X-DC patient samples will be required to confirm the efficacy and safety of the pTERC-DKC1 LV, our data support a model in which near-physiological dyskerin expression enables functional stabilization of HSPCs and confers a selective advantage to corrected cells. These findings suggest that GT may represent an effective therapeutic strategy for X-DC, particularly when applied at early disease stages in which telomere integrity and HSPC reserve are sufficiently preserved.

MATERIALS AND METHODS

Human CD34$^+$ cells

CD34$^+$ cells from umbilical CB samples were used as targets for introducing *DKC1* mutations in human HSPCs. CB samples from male newborns were provided by the Centro de Transfusión de la Comunidad de Madrid. In all cases, informed consents from the parents were obtained. Mononuclear CB cells were isolated by density gradient centrifugation using Ficoll-Paque (GE Healthcare), and CD34$^+$ cells were subsequently purified using the MACS CD34 MicroBead Kit (Miltenyi Biotec). Purified CD34$^+$ cells were resuspended at a concentration of 1–3 $\times$ 10^6 cells/mL in cryoprotective medium consisting of 20% fetal bovine serum (FBS; Gibco) and 10% dimethyl sulfoxide (DMSO) in StemSpan medium (StemCell Technologies) and stored in liquid nitrogen ($-$170°C). In all experiments, CD34$^+$ cells were thawed and pre-stimulated for 24–48 h in StemSpan medium supplemented with thrombopoietin (TPO), Flt-3 ligand, and stem cell factor (SCF) (100 ng/mL each), interleukin-3 (IL-3; 20 ng/mL) (all cytokines from EuroBiosciences), 1% GlutaMAX (Gibco), and 1% penicillin/streptomycin (Gibco).

In vitro hematopoietic cell cultures

Myeloid differentiation cultures were maintained under the same cytokine conditions described previously: TPO, Flt-3 ligand, SCF, and IL-3. The differentiation cultures were incubated at 37°C in a humidified atmosphere with 5% CO_2 for up to 30 days, at a cell density ranging from 3.5 $\times$ 10^5 to 7.0 $\times$ 10^5 cells/mL. Cell concentrations were monitored at least twice per week, and fresh medium was added as required to maintain this cell density. Cell counts were performed using a Neubauer hemocytometer, with viability assessed by Trypan Blue (0.4%) exclusion following a 1:10 dilution. For the clonogenic assays, 3,000 CD34$^+$ cells were resuspended in 6 mL of MethoCult methylcellulose medium (StemCell Technologies). Where indicated, MMC was added at a final concentration of 10 nM to assess damage sensitivity. Three aliquots of 1 mL were plated in SmartDish plates (StemCell Technologies) and incubated under the same conditions as liquid cultures. After 14 days, colonies were quantified using an automated colony counter (StemVision, StemCell Technologies). For the secondary clonogenic assays, colonies from each primary culture were pooled, and 1 $\times$ 10^5 cells from the pooled suspension were replated in 1 mL of fresh methylcellulose medium per well to assess replating efficiency.

Generation of *DKC1* mutations in human CD34$^+$ cells

To generate human HSPCs with *DKC1* mutations, the CRISPR-Cas9 system was used to introduce *DKC1* frameshift indels via NHEJ. Pre-stimulated CB CD34$^+$ cells (2 $\times$ 10^5) were electroporated with a RNP complex consisting of 6.0 μg of Alt-R S.p. HiFi Cas9 nuclease (Integrated DNA Technologies, IDT) and 3.2 μg of a single-guide RNA (sgRNA) targeting exon 4 of *DKC1* (IDT; sequence: 5′-CACTATACACCTCTTGCATG-3′). The sgRNA was designed *in silico* using IDT tools and exhibited on-target and off-target scores of 62 and 66, respectively. Electroporation was performed using a 4D-Nucleofector system (Lonza) with the P3 Primary Cell Kit and program DZ-100 in Nucleocuvette strips. Following electroporation, cells were allowed to recover for 15 min at 37°C in supplemented StemSpan medium.

To generate HSPCs with the clinically reported hypomorphic Thr66Ala (c.196 A>G) mutation,[13,19] an HDR approach was used. RNP complexes were assembled using Alt-R S.p. HiFi Cas9 nuclease and the single-guide RNA described previously targeting exon 4 of *DKC1*. To enable specific tracking of the hypomorphic allele by qPCR, synonymous nucleotide substitutions adjacent to the c.196 A>G site were incorporated into the donor sequence (see Figure S1). The donor cassette comprised the modified *DKC1* exon 4 flanked by homology arms of 893 bp (5′) and 1,655 bp (3′). The donor plasmid was synthesized by VectorBuilder (VectorBuilder GmbH) and packaged into adeno-associated virus serotype 6 (AAV6) by the same provider. Recombinant AAV6 preparations were supplied at titers exceeding 1 $\times$ 10^{11} genome copies (GC)/mL, as reported by the manufacturer. In this model, electroporated cells were immediately transduced with the donor AAV6 vector at a multiplicity of infection of 3,500 viral genomes per cell.

DKC1 editing efficiencies were initially assessed by Sanger sequencing. Genomic DNA was extracted 24 h post-editing using the DNeasy Blood & Tissue Kit (QIAGEN). An approximately 2.1 kb genomic fragment spanning *DKC1* exons 2 to 6 was amplified using Herculase II Fusion DNA Polymerase (Agilent) and primers listed in Table S2. PCR conditions were as follows: 95°C for 3 min; 35 cycles of 95°C for 20 s, 60°C for 30 s, and 70°C for 90 s; followed by a final extension at 70°C for 5 min. PCR products were subjected

to Sanger sequencing focusing on exon 4 to determine editing outcomes. Editing frequencies were quantified using the DECODR and ICE online tools, and mutation types were classified within the defined analysis window.

To quantify mutation frequencies (indels and c.196 A>G) in individual colonies and bulk cultures, custom qPCR and ddPCR assays were developed and applied to telomere length, gene expression, and rRNA maturation analyses. Primer and TaqMan probe sets were designed to amplify the *DKC1* exon 4 region, allowing discrimination between the c.196 A>G mutant allele and the wild-type sequence. An additional control amplicon ("total *DKC1*") targeting an intronic region downstream of exon 4 was used to quantify overall human *DKC1* gene copies (Tables S2 and S3).

qPCR was performed on a QuantStudio 5 Real-Time PCR System (Thermo Fisher Scientific) using Absolute QPCR Mix, Low ROX (Thermo Fisher Scientific), with the following cycling conditions: 95°C for 5 min, followed by 40 cycles of 95°C for 20 s, 60°C for 20 s, and 72°C for 10 s. Relative allele frequencies were calculated using the $2^{-\Delta\Delta Ct}$ method, accounting for the amplification efficiency of each amplicon. ddPCR was performed using the QX200 Droplet Digital PCR System (Bio-Rad) with the same primer/probe sets and cycling conditions as qPCR. Editing frequencies were determined from the fractional abundance of mutant droplets as reported by QuantaSoft software. Where sample availability permitted, qPCR-based estimates of *DKC1* allele frequencies were cross-validated with Sanger sequencing results.

In vivo editing analyses were performed on whole BM samples collected at the experimental endpoint. In mice with low levels of human engraftment (<10% $hCD45^+$ cells in BM), a nested PCR strategy was implemented to enhance mutation detection sensitivity. Briefly, a 2,064 bp region encompassing *DKC1* exon 4 and flanking intronic sequences was pre-amplified for 20 cycles using Herculase II polymerase and outer primers (Table S2), enriching for human *DKC1* sequences over mouse genomic DNA. A second PCR was then performed using inner primers flanking exon 4 with the following conditions: 95°C for 3 min; 35 cycles of 95°C for 20 s, 60°C for 20 s, and 70°C for 30 s; followed by a final extension at 70°C for 5 min. Resulting PCR products were analyzed by Sanger sequencing to determine exon 4 mutation status.

LVs

Two self-inactivating (SIN) LVs carrying the WT human *DKC1* cDNA were constructed. A transfer plasmid containing the human *DKC1* cDNA (1,545 bp) was generated (VectorBuilder). Five SIN LV transfer plasmids were used to generate the different vectors employed in this study: (1) pCCL.PGK.DKC1.WPRE, encoding *DKC1* under the control of the phosphoglycerate kinase (*PGK*) promoter (505 bp) and containing a mutated Woodchuck hepatitis virus post-transcriptional regulatory element (WPRE); (2) pCCL.pTERC.DKC1.WPRE, encoding *DKC1* driven by the regulatory region of the human *TERC* gene (−798 to −1 bp relative to the transcription start site; derived from a *TERC* gene construct spanning −798 to +1525 bp); (3) pCCL.PGK.EGFP.WPRE, expressing EGFP under the *PGK* promoter; (4) pCCL.pTERC.EGFP.WPRE, expressing EGFP under the *TERC* promoter; and (5) pCCL.pET.AGXT.mWPRE, encoding *AGXT* under the control of the synthetic liver-specific pET promoter. The pET-AGXT LV was used as a control in *in vivo* experiments to avoid interference of EGFP fluorescence with Flow-FISH telomere length measurements (kindly provided by Dr. María García-Bravo, CIEMAT).

Third-generation SIN LVs were produced by transient transfection of HEK 293 T cells (ATCC). Cells were co-transfected with the corresponding transfer plasmid and packaging plasmids encoding HIV-1 gag-pol, rev, and the vesicular stomatitis virus G (VSV-G) envelope protein. HEK 293 T cells were maintained in Dulbecco's Modified Eagle Medium (DMEM; Gibco) supplemented with 10% FBS (Sigma-Aldrich) and 0.5% penicillin/streptomycin, and transfected by the calcium phosphate method at approximately 70% confluence (24 h after plating). Viral supernatants were collected at 24 and 48 h post-transfection and concentrated by ultracentrifugation at 50,000 × *g* for 2 h. Functional LV titers were determined by transducing HEK 293 T cells with serial dilutions of each LV preparation and quantifying proviral integration by qPCR. Briefly, 5×10^4 HEK 293 T cells per well were plated in 24-well plates and transduced overnight with serial LV dilutions. Fourteen days later, genomic DNA was extracted using the DNeasy Blood & Tissue Kit (QIAGEN).

LV preparations used in this study exhibited functional titers ranging from 1×10^8 to 1×10^9 transducing units per milliliter. Proviral copy numbers were quantified by qPCR targeting the LV Ψ (psi) sequence and normalized to the human albumin (*hAlb*) gene (primer and probe sequences are listed in Tables S2 and S3). qPCR reactions were performed using Absolute QPCR Mix, low ROX (Thermo Fisher Scientific), on a QuantStudio 5 Real-Time PCR System (Thermo Fisher Scientific), with thermal cycling conditions consisting of 95°C for 10 min, followed by 40 cycles of 95°C for 15 s and 60°C for 1 min.

Lentiviral transduction of human $CD34^+$ cells

Pre-stimulated and, where applicable, gene-edited human $CD34^+$ cells were adjusted to a density of 1×10^6 cells/mL and plated on RetroNectin-coated plates for lentiviral transduction at the indicated multiplicity of infection. Transductions were performed in StemSpan medium supplemented with cytokines, as described previously. After 24 h, cells were washed and counted and then used for *in vitro* assays or transplanted into NSG mice for *in vivo* studies.

Xenogeneic transplantation

NOD-scid IL2Rγ ˆnull (NSG) mice (The Jackson Laboratory) were housed under specific pathogen-free conditions at 20°C–24°C on a 12 h light/12 h dark cycle, with *ad libitum* access to food and drinking water supplemented alternately with Baytril and Sulfatrim.

All animal procedures were approved by the competent authority of the Community of Madrid (project PROEX 156.2/21) and conducted in accordance with Spanish and European regulations (Spanish Royal Decree 53/2013 and Law 6/2003; European Directive 2010/63/EU). For the xenogeneic transplantation of human hematopoietic cells, 8-week-old NSG mice received a sublethal dose of 1.5 Gy X-ray irradiation (Philips MG324) 24 h prior to intravenous infusion of human cells. Each mouse was transplanted with 5×10^5 viable human $CD34^+$ cells, counted immediately before injection.

Telomere length measurements of *in vitro* cultured cells

Telomere length in *in vitro* cultured cells was measured using qPCR-based approaches. For cells maintained in liquid cultures, the telomere length was determined using the Human Telomere Length Quantification Kit (ScienceCell), following the manufacturer's instructions. This assay amplifies telomeric repeats (telomere primer set) and a single-copy reference sequence located on chromosome 17 (SCR primer set) for normalization. Data were analyzed using the $2^{-\Delta\Delta Ct}$ method, accounting for primer efficiencies. A reference genomic DNA sample with a known average telomere length of 13.2 kb was included as a calibrator to convert ΔΔCt values into estimated telomere length per chromosome.

For cells derived from methylcellulose colonies, genomic DNA was obtained by direct lysis of individual colonies. Colonies were lysed in 20 μL of buffer containing 0.3 mM Tris-HCl (pH 7.5), 0.6 mM $CaCl_2$, 1.5% glycerol, 0.675% Tween 20, and 0.3 mg/mL proteinase K, followed by incubation at 65°C for 30 min, 90°C for 10 min, and cooling to 4°C. Five microliters of each lysate were used for qPCR with FastStart SYBR Green Master (Rox; Roche). Telomere length in colony-derived samples was assessed using primers specific for telomeric repeats and the human β-globin (*HBB*) single-copy gene (Table S2). The telomere-to-single-copy gene (T/S) ratio was calculated using the $2^{-\Delta\Delta Ct}$ method. The qPCR program consisted of an initial denaturation at 95°C for 10 min, followed by two amplification stages: Stage 1, 5 cycles of 95°C for 15 s, 53°C for 20 s, and 68°C for 30 s; stage 2, 35 cycles of 95°C for 20 s, 57°C for 20 s, and 73°C for 45 s. Colony-derived samples from unedited, untransduced $CD34^+$ cells were used as controls for telomere attrition. Given the variable DNA yield from individual colonies and the harsh lysis conditions, stringent quality control criteria were applied to validate qPCR results. Samples were included in the analysis only if all of the following criteria were met: telomere Ct > 9.0; *HBB* Ct < 28.0; and a Ct difference (Ct_telomere − Ct_HBB) < 12.5 cycles. All qPCR reactions were performed in technical triplicates, and only samples meeting all criteria were included in downstream analyses.

Telomerase activity assay

The telomerase activity was measured using the TRAPeze Telomerase Detection Kit (Sigma-Aldrich) according to the manufacturer's instructions. Cell lysates corresponding to 1×10^6 cells were analyzed, and telomerase activity was quantified using the telomeric repeat amplification protocol (TRAP). TRAP products were normalized to the internal amplification control provided in the kit. Telomerase extension products were quantified by real-time PCR using a QuantStudio 5 system (Thermo Fisher Scientific), following the kit protocol. Telomerase activity is reported as attomoles of telomeric repeats synthesized per nanogram of total protein, normalized to the internal control.

Gene expression and rRNA maturation assays

Total RNA was extracted from *in vitro* cultured human $CD34^+$ cells using the RNeasy Plus Mini Kit (QIAGEN). Complementary DNA (cDNA) was synthesized from 1 μg of total RNA using SuperScript IV Reverse Transcriptase (Thermo Fisher Scientific) and random hexamer primers. Relative mRNA expression levels of *DKC1*, *TERT*, and *TERC* (core components of the telomerase complex), as well as additional genes associated with telomere maintenance, cell-cycle regulation, and cellular stress responses (as shown in Figure 4), were quantified by SYBR Green-based qPCR using gene-specific primers (Table S2). qPCR reactions were performed on a QuantStudio 5 Real-Time PCR System (Thermo Fisher Scientific) under the following cycling conditions: 95°C for 10 min, followed by 40 cycles of 95°C for 20 s, 58.5°C for 30 s, and 68°C for 15 s. Expression of each target gene was normalized to the housekeeping gene *GAPDH*, and relative expression levels were calculated using the $2^{-\Delta\Delta Ct}$ method.

Ribosomal pre-rRNA maturation was assessed by quantitative analysis of specific processing intermediates using qPCR. The following pre-rRNA amplicons were analyzed: 5′ETS–18S, 18S–ITS1, 5.8S–5′ITS2, and 3′ITS2–28S (Figure S1; primer sequences listed in Table S2). In parallel, total levels of mature 18S, 5.8S, and 28S rRNA were measured. Transcript abundance was normalized to *GAPDH* expression as a global transcriptional control, while 5S rRNA levels were used as a ribosomal internal reference for normalization of pre-rRNA maturation intermediates. rRNA maturation analyses were performed using SYBR FastStart Universal SYBR Green Master (Rox; Roche) on a QuantStudio 5 system, with an initial denaturation at 95°C for 5 min, followed by 40 cycles of 95°C for 10 s, 60°C for 30 s, and 69°C for 10 s. Relative abundance changes were calculated using the $2^{-\Delta\Delta Ct}$ method, taking into account the amplification efficiency of each primer set. The 41S/21S-21S-C maturation fraction was defined as the difference in relative abundance between the 18S-ITS1 and 5′ETS-18S amplicons, reflecting the processing of 41S precursors into 21S/21S-C intermediates. This parameter provides a quantitative measure of the efficiency of late steps in 18S rRNA maturation under physiological and perturbed conditions, as previously described[38,65] (see Table S1).

Western blot analyses

To evaluate the ectopic expression of human dyskerin mediated by *DKC1* LVs, transduced mouse NIH/3T3 cells were lysed in Pierce RIPA buffer (Thermo Fisher Scientific) supplemented with 1× PhosSTOP (Roche) and 1× Complete protease inhibitor cocktail (Roche). Lysates were clarified by centrifugation, and protein concentration was determined using the Bio-Rad Protein

Assay (500–0006; Bio-Rad). Equal amounts of protein (40 μg) were resolved on Mini-PROTEAN TGX Stain-Free gels (Bio-Rad) using a PowerPac HC high-current power supply (Bio-Rad) and transferred onto Trans-Blot Turbo Mini-size nitrocellulose membranes (Bio-Rad) using a Trans-Blot Turbo Transfer System (Bio-Rad). Membranes were probed with a rabbit anti-dyskerin antibody (H-300; sc-48794, Santa Cruz Biotechnology) and a mouse anti-vinculin antibody (VIN-54; ab130007, Abcam). Horseradish peroxidase-conjugated goat anti-rabbit IgG secondary antibody (ab97051, Abcam) was used for detection. Immunoreactive bands were visualized using Pierce ECL Western Blotting Substrate (Thermo Fisher Scientific) and imaged on a ChemiDoc MP Imaging System (Bio-Rad). Dyskerin expression levels were normalized to vinculin.

Analysis of human hematopoietic engraftment in NSG recipient mice

Human hematopoietic engraftment was assessed by flow cytometry up to 5 months after transplantation into NSG mice. At intermediate time points, femoral BM aspirates were collected and analyzed, while at the experimental endpoint both BM and PB samples were evaluated. Human hematopoietic chimerism was determined as the percentage of human $CD45^+$ cells using a Brilliant Violet 711-conjugated anti-human CD45 antibody (BioLegend). For the analysis of HSPCs, cells were stained with an APC-conjugated anti-human CD34 antibody (BioLegend), and $hCD34^+$ cells were gated within the $hCD45^+$ population ($hCD45^+/hCD34^+$). Lineage distribution was assessed using PE-conjugated anti-human CD19, APC/Cyanine7-conjugated anti-human CD3, and PE/Cyanine7-conjugated anti-human CD33 antibodies (all from BioLegend) to identify B ($CD19^+$), T ($CD3^+$), and myeloid ($CD33^+$) populations, respectively. Mature lineage populations were gated within the $hCD45^+/hCD34^-$ compartment.

Flow-FISH telomere length analysis in human hematopoietic cells engrafted in NSG mice

The telomere lengths in engrafted human cells were measured by flow-FISH with modifications to the standard protocol.[66] Mononuclear cells isolated from BM or PB were stained with anti-human CD45 (Brilliant Violet 711) and anti-human CD34 (Brilliant Violet 421) antibodies (BioLegend) to identify human leukocytes and $CD34^+$ subsets. Following surface staining, cells were fixed and permeabilized using the BD Cytofix/Cytoperm Fixation/Permeabilization Kit (BD Biosciences) and incubated overnight at 37°C with hybridization solution containing a fluorescently labeled telomeric probe (200 μM). After hybridization, cells were washed twice in PBS supplemented with 0.1% albumin and counterstained with propidium iodide (PI) to assess DNA content and enable cell-cycle discrimination.

Telomere length measurements were performed on $hCD45^+$ and $hCD34^+$ cell populations gated in the G0/G1 phase (see Figure S5 for the flow cytometry gating strategy) using a BD LSRFortessa flow cytometer (BD Biosciences). As a negative control, samples were processed under identical conditions in the absence of the telomeric probe. Where sample availability permitted, analyses were performed in duplicate, acquiring between 5×10^5 and 1×10^6 total events per sample. Quality thresholds for inclusion in the analysis were set at a minimum of 35,000 $hCD45^+$ events and 500 $hCD34^+$ events with detectable telomeric probe signal. To evaluate telomere length dynamics over the course of transplantation, telomere length values obtained at each time point were normalized to the corresponding value measured in the same cell population prior to xenotransplantation. In addition, telomere length variations in mouse BM cells from the same transplanted animals were recorded in all cases as an internal technical control.

Statistical analysis

Flow cytometry data were analyzed using FlowJo v.10 and FCS Express v.7 (De Novo Software). ddPCR data were analyzed using QuantaSoft v.1.7.4.0917 (Bio-Rad) in absolute quantification mode. Statistical analyses were performed using GraphPad Prism 10. Data are presented as mean ± standard error of the mean (SEM) or as median (range), as appropriate. Comparisons among three or more groups were performed using one-way or two-way ANOVA. Fisher's LSD post-hoc test was used for planned pairwise comparisons, and Tukey's, Bonferroni's, or Dunnett's corrections were applied for multiple comparisons, as indicated. A p value <0.05 was considered statistically significant. Statistical significance is indicated in figures as $*p < 0.05$, $**p < 0.01$, $***p < 0.001$, $****p < 0.0001$; ns, not significant.

DATA AND CODE AVAILABILITY

All data supporting the findings of this study are included in the main article and its supplemental information. Raw data, including sequencing files, gene-editing donor sequences, vector sequences, and image datasets, are available from the corresponding authors upon reasonable request.

ACKNOWLEDGMENTS

The authors thank the Centro de Transfusión de la Comunidad de Madrid for providing human umbilical cord blood samples. We are grateful to Rebeca Sánchez and Omaira Alberquilla from the Flow Cytometry Unit of CIEMAT for expert technical assistance in flow cytometry. We also acknowledge Montserrat de Castro, Montserrat Aldea, Aurora de la Cal, Soledad Moreno, Sergio Losada, María Jesús Arias, Eveyanira Piñero, and María Carmen Sánchez from the administrative department and the Research Management Office of the Biomedical Innovation Unit at CIEMAT for their continuous support with project coordination, regulatory procedures, sample shipment, and maintenance of biological stocks. This work was supported by the DC-INTHERGENE project (PID2021-125077OB-C22) and the TEL-REVOLUTION project (PID2024-161554OB-C22), both funded by the Ministerio de Ciencia, Innovación y Universidades. Open access funding was provided through an institutional agreement.

AUTHOR CONTRIBUTIONS

Investigation and formal analysis, D.L.-A.; investigation, M.L.L.; methodology and investigation, S.N.; methodology and investigation, L.S.; methodology and investigation, R.P.; conceptualization, writing – review and editing, and funding acquisition, J.A.B.; conceptualization, project administration, funding acquisition, and writing – review and editing, G.G.; conceptualization, investigation, formal analysis, methodology, writing – original draft, writing – review and editing, supervision, and funding acquisition, N.W.M.

DECLARATION OF INTERESTS

N.W.M. and co-authors are inventors on a patent application related to lentiviral vectors for dyskerin gene therapy (including pTERC-DKC1-based constructs), filed by their institutions.

DECLARATION OF GENERATIVE AI AND AI-ASSISTED TECHNOLOGIES IN THE WRITING PROCESS

During the preparation of this work the author(s) used Grok (built by xAI), ChatGPT (OpenAI), and DeepL Translate in order to draft parts of the text, improve language and style, and assist with translation. After using these tools/services, the author(s) reviewed and edited the content as needed and take(s) full responsibility for the content of the publication.

SUPPLEMENTAL INFORMATION

Supplemental information can be found online at https://doi.org/10.1016/j.omta.2026.201811.

REFERENCES

1. Kam, M.L.W., Nguyen, T.T.T., and Ngeow, J.Y.Y. (2021). Telomere biology disorders. npj Genom. Med. *6*, 36. https://doi.org/10.1038/s41525-021-00198-5.
2. Grill, S., and Nandakumar, J. (2021). Molecular mechanisms of telomere biology disorders. J. Biol. Chem. *296*, 100064. https://doi.org/10.1074/jbc.REV120.014017.
3. Guenechea, G., and Meza, N.W. (2024). The gray boundaries of aberrant shortening of the cellular timekeepers' edges. EMBO Mol. Med. *16*, 2276–2278. https://doi.org/10.1038/S44321-024-00122-1.
4. Tummala, H., Walne, A., and Dokal, I. (2022). The biology and management of dyskeratosis congenita and related disorders of telomeres. Expert Rev. Hematol. *15*, 685–696. https://doi.org/10.1080/17474086.2022.2108784.
5. Revy, P., Kannengiesser, C., and Bertuch, A.A. (2023). Genetics of human telomere biology disorders Genetics of human telomere biology disorders. Nat. Rev. Genet. *24*, 86–108. https://doi.org/10.1038/s41576-022-00527-z.
6. Garus, A., and Autexier, C. (2021). Dyskerin: an essential pseudouridine synthase with multifaceted roles in ribosome biogenesis, splicing, and telomere maintenance. RNA *27*, 1441–1458. https://doi.org/10.1261/RNA.078953.121.
7. Udroiu, I., Marinaccio, J., and Sgura, A. (2022). Many Functions of Telomerase Components: Certainties, Doubts, and Inconsistencies. Int. J. Mol. Sci. *23*, 15189. https://doi.org/10.3390/IJMS232315189.
8. Romaniuk, A., Paszel-Jaworska, A., Totoń, E., Lisiak, N., Hołysz, H., Królak, A., Grodecka-Gazdecka, S., and Rubiś, B. (2019). The non-canonical functions of telomerase: to turn off or not to turn off. Mol. Biol. Rep. *46*, 1401–1411. https://doi.org/10.1007/S11033-018-4496-X.
9. Dokal, I., Tummala, H., and Vulliamy, T. (2022). Inherited bone marrow failure in the pediatric patient. Blood *140*, 556–570. https://doi.org/10.1182/blood.2020006481.
10. Blasco, M.A. (2005). Telomeres and human disease: ageing, cancer and beyond. Nat. Rev. Genet. *6*, 611–622. https://doi.org/10.1038/nrg1656.
11. Vinayagamurthy, S., Ganguly, A., and Chowdhury, S. (2020). Extra-telomeric impact of telomeres: Emerging molecular connections in pluripotency or stemness. J. Biol. Chem. *295*, 10245–10254. https://doi.org/10.1074/JBC.REV119.009710.
12. AlSabbagh, M.M. (2020). Dyskeratosis congenita: a literature review. J. Dtsch. Dermatol. Ges. *18*, 943–967. https://doi.org/10.1111/ddg.14268.
13. Glousker, G., Touzot, F., Revy, P., Tzfati, Y., and Savage, S.A. (2015). Unraveling the Pathogenesis of Hoyeraal-Hreidarsson Syndrome, a Complex Telomere Biology Disorder. Br. J. Haematol. *170*, 457–471. https://doi.org/10.1111/BJH.13442.
14. Niewisch, M.R., Giri, N., McReynolds, L.J., Alsaggaf, R., Bhala, S., Alter, B.P., and Savage, S.A. (2022). Disease progression and clinical outcomes in telomere biology disorders. Blood *139*, 1807–1819. https://doi.org/10.1182/blood.2021013523.
15. Niewisch, M.R., and Savage, S.A. (2019). An update on the biology and management of dyskeratosis congenita and related telomere biology disorders. Expert Rev. Hematol. *12*, 1037–1052. https://doi.org/10.1080/17474086.2019.1662720.
16. Bluteau, O., Sebert, M., Leblanc, T., Peffault de Latour, R., Quentin, S., Lainey, E., Hernandez, L., Dalle, J.H., Sicre de Fontbrune, F., Lengline, E., et al. (2018). A landscape of germline mutations in a cohort of inherited bone marrow failure patients. Blood *131*, 717–732. https://doi.org/10.1182/blood-2017-09-806489.
17. Home - OMIM https://www.omim.org/.
18. Heiss, N.S., Knight, S.W., Vulliamy, T.J., Klauck, S.M., Wiemann, S., Mason, P.J., Poustka, A., and Dokal, I. (1998). X-linked dyskeratosis congenita is caused by mutations in a highly conserved gene with putative nucleolar functions. Nat. Genet. *19*, 32–38. https://doi.org/10.1038/NG0598-32.
19. Vulliamy, T.J., Marrone, A., Knight, S.W., Walne, A., Mason, P.J., and Dokal, I. (2006). Mutations in dyskeratosis congenita: their impact on telomere length and the diversity of clinical presentation. Blood *107*, 2680–2685. https://doi.org/10.1182/BLOOD-2005-07-2622.
20. Pederiva, C., Trevisan, D.M., Peirasmaki, D., Chen, S., Savage, S.A., Larsson, O., Ule, J., Baranello, L., Agostini, F., and Farnebo, M. (2023). Control of protein synthesis through mRNA pseudouridylation by dyskerin. Sci. Adv. *9*, eadg1805. https://doi.org/10.1126/sciadv.adg1805.
21. Fiesco-Roa, M., García-de Teresa, B., Leal-Anaya, P., van 't Hek, R., Wegman-Ostrosky, T., Frías, S., and Rodríguez, A. (2022). Fanconi anemia and dyskeratosis congenita/telomere biology disorders: Two inherited bone marrow failure syndromes with genomic instability. Front. Oncol. *12*, 949435. https://doi.org/10.3389/FONC.2022.949435.
22. Nourbakhsh, M., Golestani, A., Zahrai, M., Modarressi, M.H., Malekpour, Z., and Karami-Tehrani, F. (2010). Androgens stimulate telomerase expression, activity and phosphorylation in ovarian adenocarcinoma cells. Mol. Cell. Endocrinol. *330*, 10–16. https://doi.org/10.1016/j.mce.2010.07.021.
23. Khincha, P.P., Wentzensen, I.M., Giri, N., Alter, B.P., and Savage, S.A. (2014). Response to androgen therapy in patients with dyskeratosis congenita. Br. J. Haematol. *165*, 349–357. https://doi.org/10.1111/bjh.12748.
24. Barbaro, P., and Vedi, A. (2016). Survival after Hematopoietic Stem Cell Transplant in Patients with Dyskeratosis Congenita: Systematic Review of the Literature. Biol. Blood Marrow Transplant. *22*, 1152–1158. https://doi.org/10.1016/j.bbmt.2016.03.001.
25. Elmahadi, S., Muramatsu, H., and Kojima, S. (2016). Allogeneic hematopoietic stem cell transplantation for dyskeratosis congenita. Curr. Opin. Hematol. *23*, 501–507. https://doi.org/10.1097/MOH.0000000000000290.
26. Fioredda, F., Iacobelli, S., Korthof, E.T., Knol, C., van Biezen, A., Bresters, D., Veys, P., Yoshimi, A., Fagioli, F., Mats, B., et al. (2018). Outcome of haematopoietic stem cell transplantation in dyskeratosis congenita. Br. J. Haematol. *183*, 110–118. https://doi.org/10.1111/BJH.15495.
27. Georges, G.E., Doney, K., and Storb, R. (2018). Severe aplastic anemia: allogeneic bone marrow transplantation as first-line treatment. Blood Adv. *2*, 2020–2028. https://doi.org/10.1182/bloodadvances.2018021162.
28. Dimitrov, M., Merkle, S., Cao, Q., Tryon, R.K., Vercellotti, G.M., Holtan, S.G., Kao, R.L., Srikanthan, M., Terezakis, S.A., Tolar, J., and Ebens, C.L. (2024). Allogeneic Hematopoietic Cell Transplant For Bone Marrow Failure or Myelodysplastic Syndrome in Dyskeratosis Congenita/Telomere Biology Disorders: Single-Center, Single-Arm, Open-Label Trial of Reduced-Intensity Conditioning Without Radiation. Transplant. Cell. Ther *30*, 1005.e1–1005.e17. https://doi.org/10.1016/j.jtct.2024.07.007.
29. Czechowicz, A., Sevilla, J., Booth, C., Agarwal, R., Zubicaray, J., Río, P., Navarro, S., Chetty, K., O'Toole, G., Xu-Bayford, J., et al. (2022). Lentiviral-mediated Gene Therapy for Patients with Fanconi Anemia [Group A]: Updated Results from Global RP-L102 Clinical Trials. Blood *140*, 10646–10647. https://doi.org/10.1182/BLOOD-2022-168342.
30. Río, P., Zubicaray, J., Navarro, S., Gálvez, E., Sánchez-Domínguez, R., Nicoletti, E., Sebastián, E., Rothe, M., Pujol, R., Bogliolo, M., et al. (2024). Haematopoietic gene therapy of non-conditioned patients with Fanconi anaemia-A: results from open-label phase 1/2 (FANCOLEN-1) and long-term clinical trials. Lancet *404*, 2584–2592. https://doi.org/10.1016/S0140-6736(24)01880-4.
31. Río, P., Navarro, S., Wang, W., Sánchez-Domínguez, R., Pujol, R.M., Segovia, J.C., Bogliolo, M., Merino, E., Wu, N., Salgado, R., et al. (2019). Successful engraftment of gene-corrected hematopoietic stem cells in non-conditioned patients with

Fanconi anemia. Nat. Med. *25*, 1396–1401. https://doi.org/10.1038/s41591-019-0550-z.

32. McKenna, E., Traganos, F., Zhao, H., and Darzynkiewicz, Z. (2012). Persistent DNA damage caused by low levels of mitomycin C induces irreversible cell senescence. Cell Cycle *11*, 3132–3140. https://doi.org/10.4161/CC.21506.

33. Snodgrass, R.G., Collier, A.C., Coon, A.E., and Pritsos, C.A. (2010). Mitomycin C inhibits ribosomal RNA: a novel cytotoxic mechanism for bioreductive drugs. J. Biol. Chem. *285*, 19068–19075. https://doi.org/10.1074/JBC.M109.040477.

34. Kampen, K.R., Sulima, S.O., Vereecke, S., and de Keersmaecker, K. (2020). Hallmarks of ribosomopathies. Nucleic Acids Res. *48*, 1013–1028. https://doi.org/10.1093/NAR/GKZ637.

35. Orgebin, E., Lamoureux, F., Isidor, B., Charrier, C., Ory, B., Lézot, F., and Baud'huin, M. (2020). Ribosomopathies: New Therapeutic Perspectives. Cells *9*, 2080. https://doi.org/10.3390/cells9092080.

36. Penzo, M., Rocchi, L., Brugiere, S., Carnicelli, D., Onofrillo, C., Couté, Y., Brigotti, M., and Montanaro, L. (2015). Human ribosomes from cells with reduced dyskerin levels are intrinsically altered in translation. FASEB J. *29*, 3472–3482. https://doi.org/10.1096/fj.15-270991.

37. López, J., and Blanco, S. (2024). Exploring the role of ribosomal RNA modifications in cancer. Curr. Opin. Genet. Dev. *86*, 102204. https://doi.org/10.1016/J.GDE.2024.102204.

38. Azouzi, C., Jaafar, M., Dez, C., Abou Merhi, R., Lesne, A., Henras, A.K., and Gadal, O. (2021). Coupling Between Production of Ribosomal RNA and Maturation: Just at the Beginning. Front. Mol. Biosci. *8*, 778778. https://doi.org/10.3389/fmolb.2021.778778.

39. Montanaro, L., Brigotti, M., Clohessy, J., Barbieri, S., Ceccarelli, C., Santini, D., Taffurelli, M., Calienni, M., Teruya-Feldstein, J., Trerè, D., et al. (2006). Dyskerin expression influences the level of ribosomal RNA pseudo-uridylation and telomerase RNA component in human breast cancer. J. Pathol. *210*, 10–18. https://doi.org/10.1002/PATH.2023.

40. Hiregange, D.G., Rivalta, A., Yonath, A., Zimmerman, E., Bashan, A., and Yonath, H. (2022). Mutations in RPS19 may affect ribosome function and biogenesis in Diamond Blackfan anemia. FEBS Open Bio *12*, 1419–1434. https://doi.org/10.1002/2211-5463.13444.

41. Giménez, Y., Palacios, M., Sánchez-Domínguez, R., Zorbas, C., Peral, J., Puzik, A., Ugalde, L., Alberquilla, O., Villanueva, M., Río, P., et al. (2024). Lentivirus-mediated gene therapy corrects ribosomal biogenesis and shows promise for Diamond Blackfan anemia. JCI Insight *9*, e171650. https://doi.org/10.1172/JCI.INSIGHT.171650.

42. Poletti, V., and Mavilio, F. (2021). Designing Lentiviral Vectors for Gene Therapy of Genetic Diseases. Viruses *13*, 1526. https://doi.org/10.3390/V13081526.

43. Shukla, S., Schmidt, J.C., Goldfarb, K.C., Cech, T.R., and Parker, R. (2016). Inhibition of telomerase RNA decay rescues telomerase deficiency caused by dyskerin or PARN defects. Nat. Struct. Mol. Biol. *23*, 286–292. https://doi.org/10.1038/nsmb.3184.

44. Wong, J.M.Y., and Collins, K. (2006). Telomerase RNA level limits telomere maintenance in X-linked dyskeratosis congenita. Genes Dev. *20*, 2848–2858. https://doi.org/10.1101/GAD.1476206.

45. Nagpal, N., Wang, J., Zeng, J., Lo, E., Moon, D.H., Luk, K., Braun, R.O., Burroughs, L.M., Keel, S.B., Reilly, C., et al. (2020). Small-Molecule PAPD5 Inhibitors Restore Telomerase Activity in Patient Stem Cells. Cell Stem Cell *26*, 896–909.e8. https://doi.org/10.1016/j.stem.2020.03.016.

46. Jung, M., Dunbar, C.E., and Winkler, T. (2015). Modeling Human Bone Marrow Failure Syndromes Using Pluripotent Stem Cells and Genome Engineering. Mol. Ther. *23*, 1832–1842. https://doi.org/10.1038/mt.2015.180.

47. Kohn, D.B., Chen, Y.Y., and Spencer, M.J. (2023). Successes and challenges in clinical gene therapy. Gene Ther. *30*, 738–746. https://doi.org/10.1038/s41434-023-00390-5.

48. Tucci, F., Galimberti, S., Naldini, L., Valsecchi, M.G., and Aiuti, A. (2022). A systematic review and meta-analysis of gene therapy with hematopoietic stem and progenitor cells for monogenic disorders. Nat. Commun. *13*, 1315. https://doi.org/10.1038/S41467-022-28762-2.

49. Bueren, J.A., Quintana-Bustamante, O., Almarza, E., Navarro, S., Río, P., Segovia, J.C., and Guenechea, G. (2020). Advances in the gene therapy of monogenic blood cell diseases. Clin. Genet. *97*, 89–102. https://doi.org/10.1111/CGE.13593.

50. Río, P., Navarro, S., Guenechea, G., Sánchez-Domínguez, R., Lamana, M.L., Yañez, R., Casado, J.A., Mehta, P.A., Pujol, M.R., Surrallés, J., et al. (2017). Engraftment and in vivo proliferation advantage of gene-corrected mobilized CD34+ cells from Fanconi anemia patients. Blood *130*, 1535–1542. https://doi.org/10.1182/BLOOD-2017-03-774174.

51. Goldman, F.D., Aubert, G., Klingelhutz, A.J., Hills, M., Cooper, S.R., Hamilton, W.S., Schlueter, A.J., Lambie, K., Eaves, C.J., and Lansdorp, P.M. (2008). Characterization of primitive hematopoietic cells from patients with dyskeratosis congenita. Blood *111*, 4523–4531. https://doi.org/10.1182/blood-2007-10-120204.

52. Kirwan, M., Vulliamy, T., Beswick, R., Walne, A.J., Casimir, C., and Dokal, I. (2008). Circulating haematopoietic progenitors are differentially reduced amongst subtypes of dyskeratosis congenita. Br. J. Haematol. *140*, 719–722. https://doi.org/10.1111/J.1365-2141.2008.06991.X.

53. Nelson, A.S., Marsh, R.A., Myers, K.C., Davies, S.M., Jodele, S., O'Brien, T.A., and Mehta, P.A. (2016). A Reduced-Intensity Conditioning Regimen for Patients with Dyskeratosis Congenita Undergoing Hematopoietic Stem Cell Transplantation. Biol. Blood Marrow Transplant. *22*, 884–888. https://doi.org/10.1016/J.BBMT.2016.01.026.

54. Montanaro, L., Tazzari, P.L., and Derenzini, M. (2003). Enhanced telomere shortening in transformed lymphoblasts from patients with X linked dyskeratosis. J. Clin. Pathol. *56*, 583–586. https://doi.org/10.1136/JCP.56.8.583.

55. Fong, Y.W., Ho, J.J., Inouye, C., and Tjian, R. (2014). The dyskerin ribonucleoprotein complex as an OCT4/SOX2 coactivator in embryonic stem cells. eLife *3*, e03573. https://doi.org/10.7554/eLife.03573.

56. Pereboom, T.C., van Weele, L.J., Bondt, A., and MacInnes, A.W. (2011). A zebrafish model of dyskeratosis congenita reveals hematopoietic stem cell formation failure resulting from ribosomal protein-mediated p53 stabilization. Blood *118*, 5458–5465. https://doi.org/10.1182/blood-2011-04-351460.

57. Ibáñez-Cabellos, J.S., Seco-Cervera, M., Picher-Latorre, C., Pérez-Machado, G., García-Giménez, J.L., and Pallardó, F.V. (2020). Acute depletion of telomerase components DKC1 and NOP10 induces oxidative stress and disrupts ribosomal biogenesis via NPM1 and activation of the P53 pathway. Biochim. Biophys. Acta Mol. Cell Res. *1867*, 118845. https://doi.org/10.1016/j.bbamcr.2020.118845.

58. Thongon, N., Ma, F., Santoni, A., Marchesini, M., Fiorini, E., Rose, A., Adema, V., Ganan-Gomez, I., Groarke, E.M., Gutierrez-Rodrigues, F., et al. (2021). Hematopoiesis under telomere attrition at the single-cell resolution. Nat. Commun. *12*, 6850. https://doi.org/10.1038/s41467-021-27206-7.

59. Zeng, T., Lv, G., Chen, X., Yang, L., Zhou, L., Dou, Y., Tang, X., Yang, J., An, Y., and Zhao, X. (2020). CD8+ T-cell senescence and skewed lymphocyte subsets in young Dyskeratosis Congenita patients with PARN and DKC1 mutations. J. Clin. Lab. Anal. *34*, e23375. https://doi.org/10.1002/JCLA.23375.

60. Balch, S., Sekne, Z., Franco-Echevarría, E., Ludzia, P., Kretsch, R.C., Sun, W., Yu, H., Ghanim, G.E., Thorkelsson, S., Ding, Y., et al. (2025). Cryo-EM structure of human telomerase dimer reveals H/ACA RNP-mediated dimerization. Science *389*, eadr5817. https://doi.org/10.1126/SCIENCE.ADR5817.

61. Bellodi, C., McMahon, M., Contreras, A., Juliano, D., Kopmar, N., Nakamura, T., Maltby, D., Burlingame, A., Savage, S.A., Shimamura, A., and Ruggero, D. (2013). H/ACA Small RNA Dysfunctions in Disease Reveal Key Roles for Noncoding RNA Modifications in Hematopoietic Stem Cell Differentiation. Cell Rep. *3*, 1493–1502. https://doi.org/10.1016/J.CELREP.2013.04.030.

62. Gu, B.W., Apicella, M., Mills, J., Fan, J.M., Reeves, D.A., French, D., Podsakoff, G.M., Bessler, M., and Mason, P.J. (2015). Impaired Telomere Maintenance and Decreased Canonical WNT Signaling but Normal Ribosome Biogenesis in Induced Pluripotent Stem Cells from X-Linked Dyskeratosis Congenita Patients. PLoS One *10*, e0127414. https://doi.org/10.1371/JOURNAL.PONE.0127414.

63. Guerrieri, A.N., Zacchini, F., Onofrillo, C., Di Viggiano, S., Penzo, M., Ansuini, A., Gandin, I., Nobe, Y., Taoka, M., Isobe, T., et al. (2020). DKC1 Overexpression

Induces a More Aggressive Cellular Behavior and Increases Intrinsic Ribosomal Activity in Immortalized Mammary Gland Cells. Cancers (Basel) *12*, 3512–3514. https://doi.org/10.3390/CANCERS12123512.

64. Myers, K.C., Davies, S.M., Lutzko, C., Wahle, R., Grier, D.D., Aubert, G., Norris, K., Baird, D.M., Koga, M., Ko, A.C., et al. (2025). Clinical Use of ZSCAN4 for Telomere Elongation in Hematopoietic Stem Cells. NEJM Evid. *4*, EVIDoa2400252. https://doi.org/10.1056/EVIDOA2400252.

65. Dörner, K., Ruggeri, C., Zemp, I., and Kutay, U. (2023). Ribosome biogenesis factors—from names to functions. EMBO J. *42*, e112699. https://doi.org/10.15252/EMBJ.2022112699.

66. Baerlocher, G.M., Vulto, I., de Jong, G., and Lansdorp, P.M. (2006). Flow cytometry and FISH to measure the average length of telomeres (flow FISH). Nat. Protoc. *1*, 2365–2376. https://doi.org/10.1038/nprot.2006.263.

OMTA, Volume *34*

Supplemental information

The potential of gene therapy to rescue telomeric and extratelomeric defects in X-linked dyskeratosis congenita

Diego Llorente-Arroyo, María Luz Lozano, Susana Navarro, Leandro Sastre, Rosario Perona, Juan Antonio Bueren, Guillermo Guenechea, and Nestor Wenceslao Meza

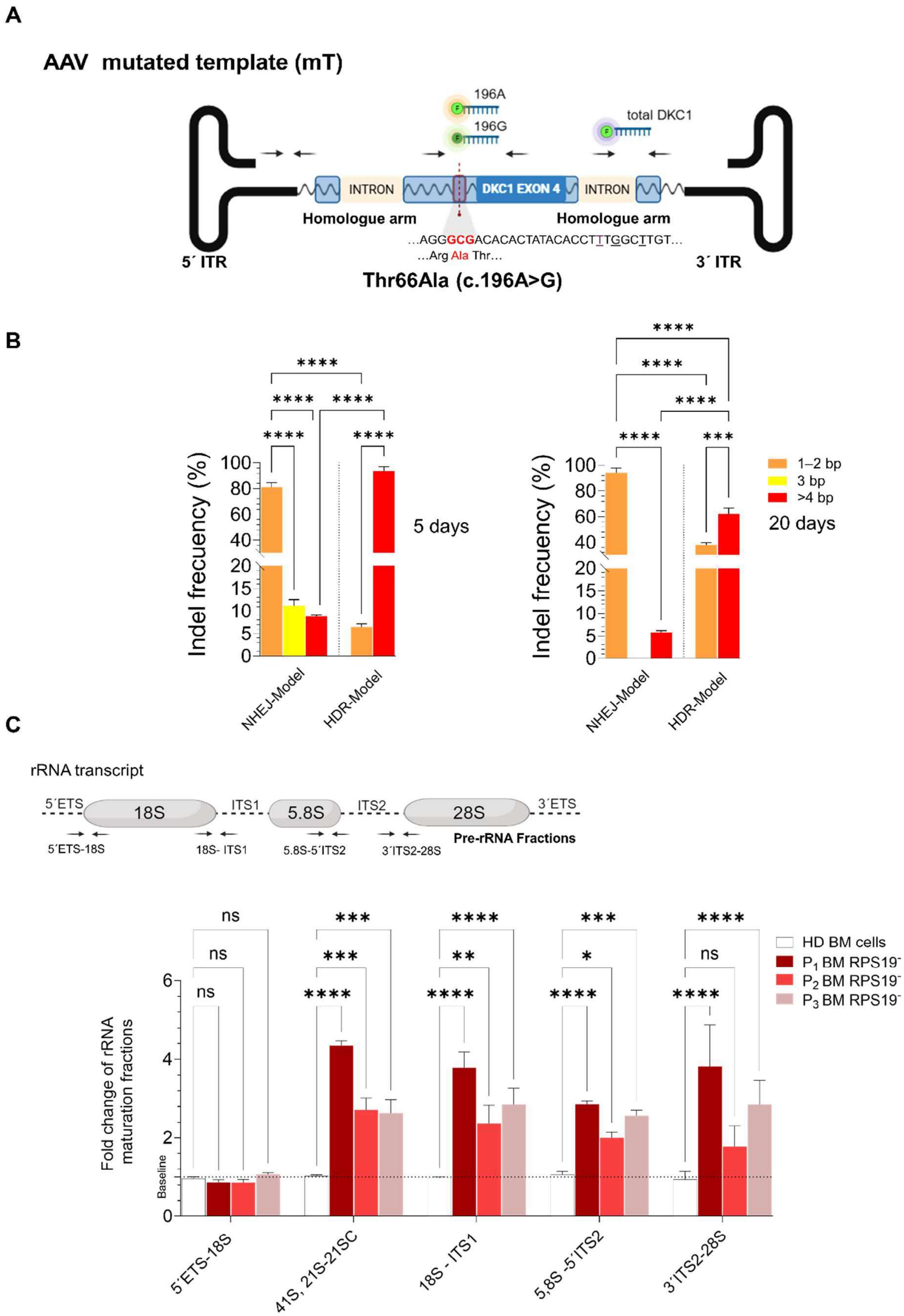
A
AAV mutated template (mT)
196A
196G
total DKC1
INTRON
DKC1 EXON 4
INTRON
Homologue arm
Homologue arm
...AGGGCGACACACTATACACCTTTGGCTTGT...
...Arg Ala Thr...
5´ ITR
3´ ITR
Thr66Ala (c.196A>G)
B
Indel frecuency (%)
5 days
20 days
1–2 bp
3 bp
>4 bp
NHEJ-Model
HDR-Model
C
rRNA transcript
5´ETS
18S
ITS1
5.8S
ITS2
28S
3´ETS
5´ETS-18S
18S- ITS1
5.8S-5´ITS2
3´ITS2-28S
Pre-rRNA Fractions
Fold change of rRNA maturation fractions
Baseline
HD BM cells
P1 BM RPS19-
P2 BM RPS19-
P3 BM RPS19-
5´ETS-18S
41S, 21S-21SC
18S - ITS1
5.8S -5´ITS2
3´ITS2-28S

Fig. S1. Generation and consequences of a hypomorphic *DKC1* mutation in human HSPCs

(A) Schematic representation of the single-stranded AAV (ssAAV) donor used to introduce the missense 196A>G (Thr66Ala) mutation into human CD34^{+} cells by homology-directed repair (HDR). Arrows indicate the positions of flanking primers used to quantify modifications within *DKC1* exon 4 by PCR. The region containing the qPCR detection probes is indicated by coloured lines (see Supplementary Tables 2 and 3 for oligonucleotide sequences). (B) Distribution of indel mutations detected after editing of *DKC1* exon 4 in the NHEJ and HDR models. Orange bars represent frameshift indels of 1-2 base pairs (non-multiples of three) predicted to generate premature stop codons. Yellow bars indicate in-frame deletions of exactly three base pairs (corresponding to the loss of one amino acid). Red bars represent larger deletions (>4 base pairs), which were grouped for clarity. For comparisons of indel frequencies between editing models, Tukey's multiple-comparison correction was applied. (C) Ribosomal RNA (rRNA) maturation analysis in edited cells assessed by RT-qPCR. The schematic at the top illustrates the immature rRNA regions recognized by each primer pair, corresponding to four distinct pre-rRNA fractions that capture successive stages of rRNA processing. A fifth fraction (41S/21S-21SC) was calculated by subtracting the 5′ETS-18S signal from the 18S-ITS1 signal, thereby reflecting an intermediate-to-late stage of rRNA maturation (see Methods and Supplementary Table 1). The graph shows the relative abundance of rRNA precursors in bone marrow samples from patients with Diamond–Blackfan anemia (P1–P3; all RPS19-deficient), used as positive controls for the rRNA maturation assay. N = 3 patients, with each sample measured in

technical triplicates. Data are shown as mean ± SEM. Statistical analysis was performed using Fisher's least significant difference (LSD) test.

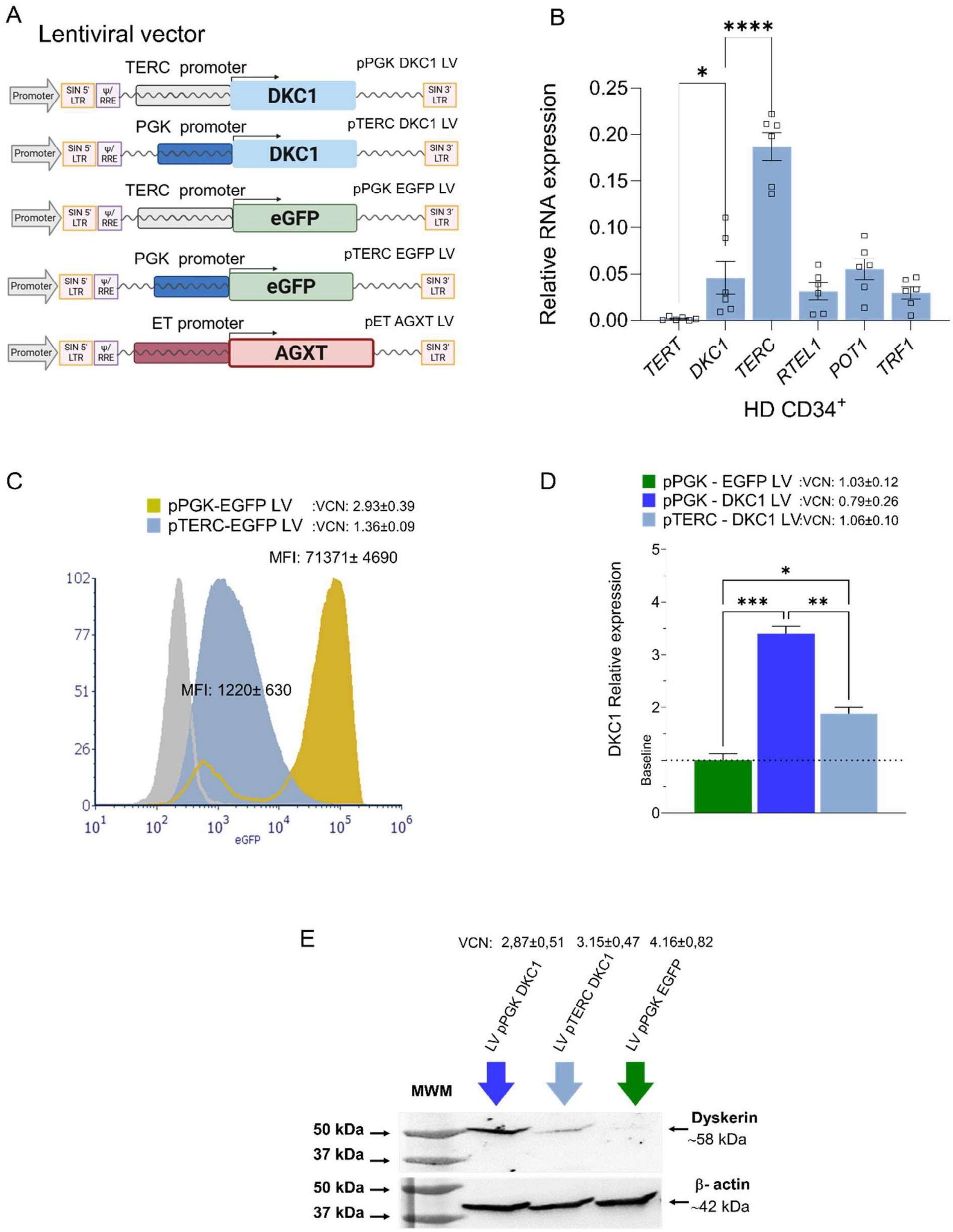

A
Lentiviral vector
TERC promoter
pPGK DKC1 LV
DKC1
PGK promoter
pTERC DKC1 LV
DKC1
TERC promoter
pPGK EGFP LV
eGFP
PGK promoter
pTERC EGFP LV
eGFP
ET promoter
pET AGXT LV
AGXT
Promoter
SIN 5' LTR
ψ/ RRE
SIN 3' LTR
B
Relative RNA expression
0.25
0.20
0.15
0.10
0.05
0.00
*
****
TERT
DKC1
TERC
RTEL1
POT1
TRF1
HD CD34+
C
pPGK-EGFP LV :VCN: 2.93±0.39
pTERC-EGFP LV :VCN: 1.36±0.09
MFI: 71371± 4690
MFI: 1220± 630
102
77
51
26
0
eGFP
D
pPGK - EGFP LV :VCN: 1.03±0.12
pPGK - DKC1 LV :VCN: 0.79±0.26
pTERC - DKC1 LV:VCN: 1.06±0.10
DKC1 Relative expression
Baseline
*
***
**
E
VCN: 2,87±0,51 3.15±0,47 4.16±0,82
LV pPGK DKC1
LV pTERC DKC1
LV pPGK EGFP
MWM
50 kDa
37 kDa
Dyskerin
~58 kDa
β- actin
~42 kDa

Fig. S2. Functional characterization of *DKC1* lentiviral vectors

(A) Schematic representation of the lentiviral vectors used to express human *DKC1*. Therapeutic vectors encoding dyskerin cDNA were constructed using either the human phosphoglycerate kinase promoter (pPGK) or a 798-bp fragment of the endogenous human *TERC* promoter (pTERC). Corresponding control vectors expressed EGFP instead of *DKC1*, and an unrelated liver-specific lentiviral vector (pET-AGXT) was used as a negative control during *in vivo* experiments (see Methods for details). (B) Relative abundance of *TERC*, *DKC1*, *TERT*, *RTEL1*, *POT1*, and *TRF1* transcripts in healthy donor $CD34^+$ cells five days after lentiviral transduction, normalized to the *GAPDH* housekeeping gene. Data represent mean ± SEM from six independent experiments (N = 6). Statistical significance was assessed using Tukey's multiple-comparison test. (C) Mean fluorescence intensity (MFI) of EGFP in healthy donor $CD34^+$ cells 20 days after transduction with a high multiplicity of infection (MOI = 100) of the promoter test vectors pPGK-EGFP (dark yellow) or pTERC-EGFP (light blue). This panel reflects the relative transcriptional strength of each promoter as assessed by EGFP expression. (D) Relative dyskerin mRNA levels in healthy donor $CD34^+$ cells 20 days after transduction with either the pPGK-DKC1 (dark blue) or pTERC-DKC1 (light blue) lentiviral vectors, normalized to levels measured in cells transduced with the control pPGK-EGFP vector (green). Data represent mean ± SEM from two independent experiments (N = 2). Tukey's multiple-comparison test was used for statistical analysis. (E) Western blot analysis of dyskerin protein expression driven by the pPGK-DKC1 and pTERC-DKC1 lentiviral vectors in transduced NIH-3T3 cells. A band of approximately 58 kDa corresponding to human dyskerin is detected in cells transduced with either *DKC1* vector and is absent in untransduced controls. The vector copy number per cell (VCN/cell) measured for

each condition is indicated in panels C–E, demonstrating comparable transduction levels across experimental groups.

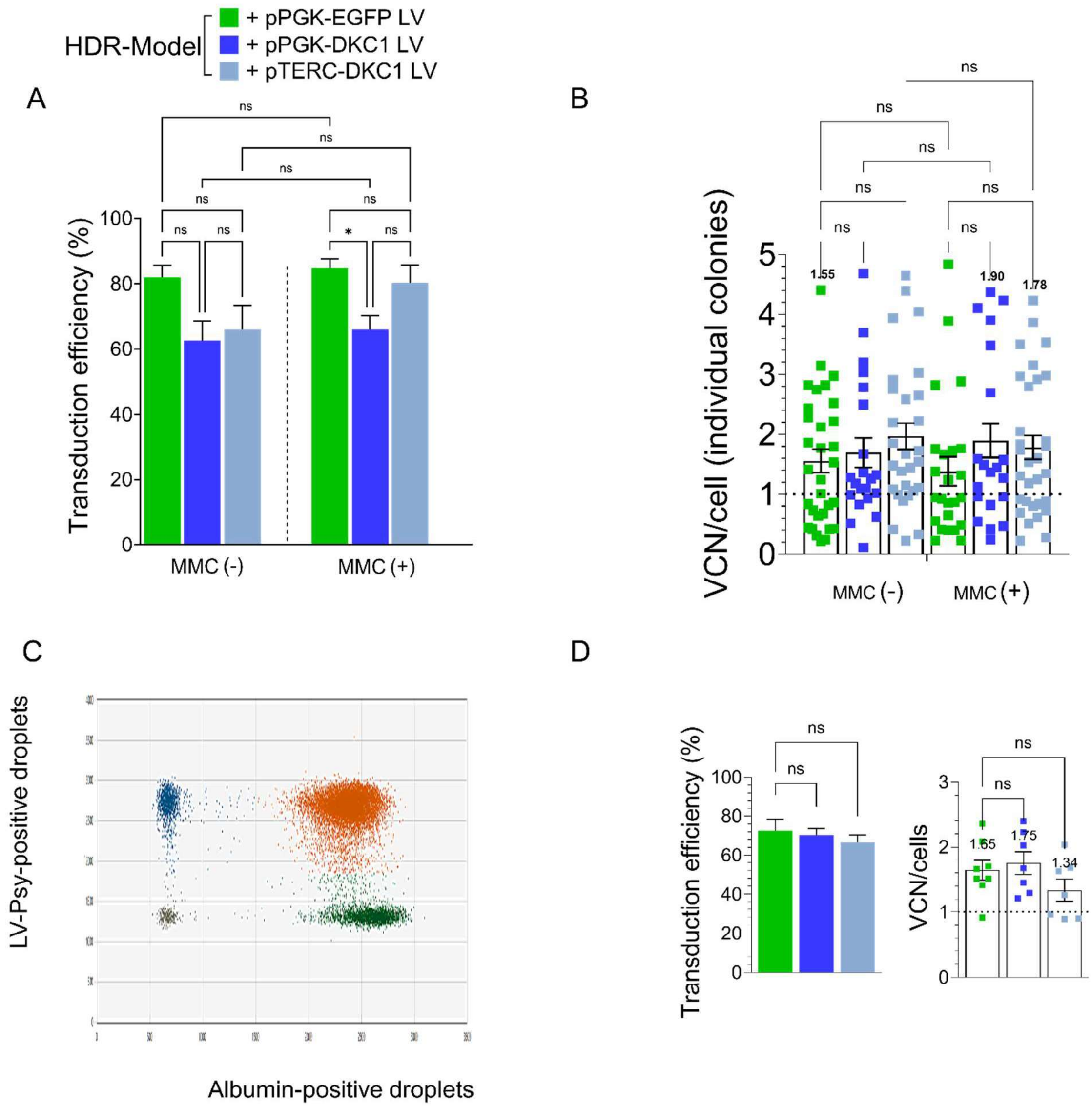

Fig. S3. Lentiviral transduction efficiency in X-DC-like HSPCs

(A) Percentage of hematopoietic colonies positive for lentiviral proviral integration across the experimental conditions shown in Fig. 3. Data represent mean ± SEM from four independent experiments (N = 4). (B) Vector copy number per cell (VCN/cell) measured in individual colonies derived from the experiments shown in Fig. 3. Each bar represents the

mean VCN calculated from 20-40 colonies per condition, illustrating the distribution of lentiviral integration events. (C) Two-dimensional representation of VCN in bulk cultures, measured by droplet digital PCR (ddPCR) after 20 days of standard liquid culture for each experimental group. (D) Lentiviral transduction efficiency and vector copy number in 20-day liquid cultures corresponding to the experimental conditions shown in Fig. 4. Left: percentage of lentivirus-transduced cells (transduction rate). Right: corresponding VCN per cell measured in bulk culture. These paired analyses allow comparison of transduction efficiency and vector copy stability over time. Statistical comparisons between groups in panel D were performed using Tukey's multiple-comparison test.

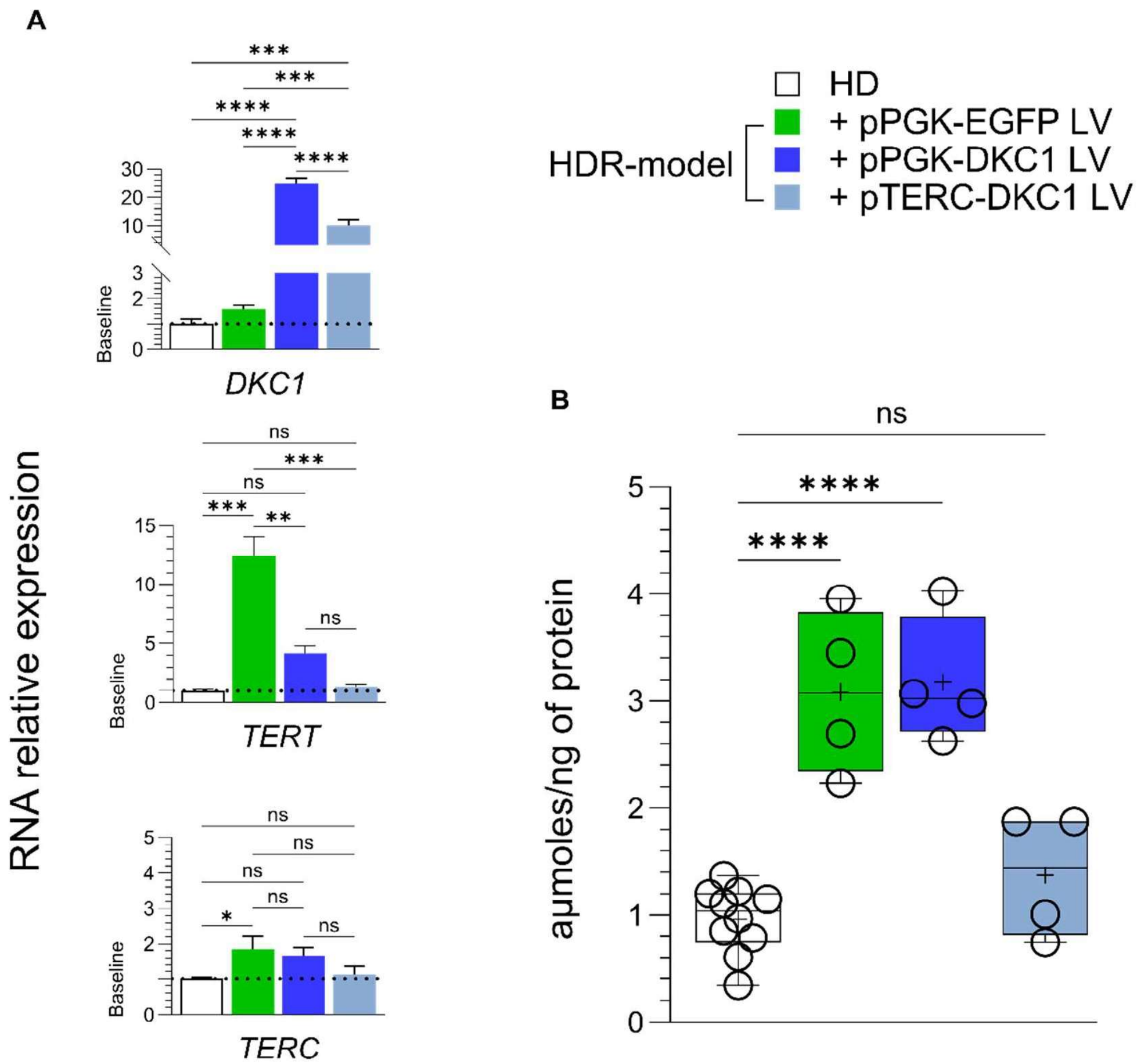

Fig. S4. Regulation of telomerase-related components in X-DC-like HSPCs after *DKC1* gene transfer. (A) Expression levels of *DKC1*, *TERT*, and *TERC* transcripts in X-DC–like CD34$^+$ cells transduced with the indicated lentiviral vectors: pPGK-EGFP (green bars; non-therapeutic control), pPGK-DKC1 (dark blue bars), or pTERC-DKC1 (light blue bars). Unedited and untransduced CD34$^+$ cells from healthy donors (HD; white bars) were cultured in parallel and used as baseline controls. RNA levels were quantified five days post-transduction, at a time point when cultures contained approximately 30–60% CD34$^+$ progenitor cells. Data represent mean ± SEM from three independent experiments (N = 3). (B) Telomerase activity measured in cell lysates from the same experimental groups shown

in panel A, expressed as units of telomere repeat amplification protocol (TRAP) product per nanogram of protein. Sample sizes were N = 4 for each transduced group and N = 8 for the untreated HD control group. Statistical comparisons between groups were performed using Fisher's least significant difference (LSD) multiple-comparison test.

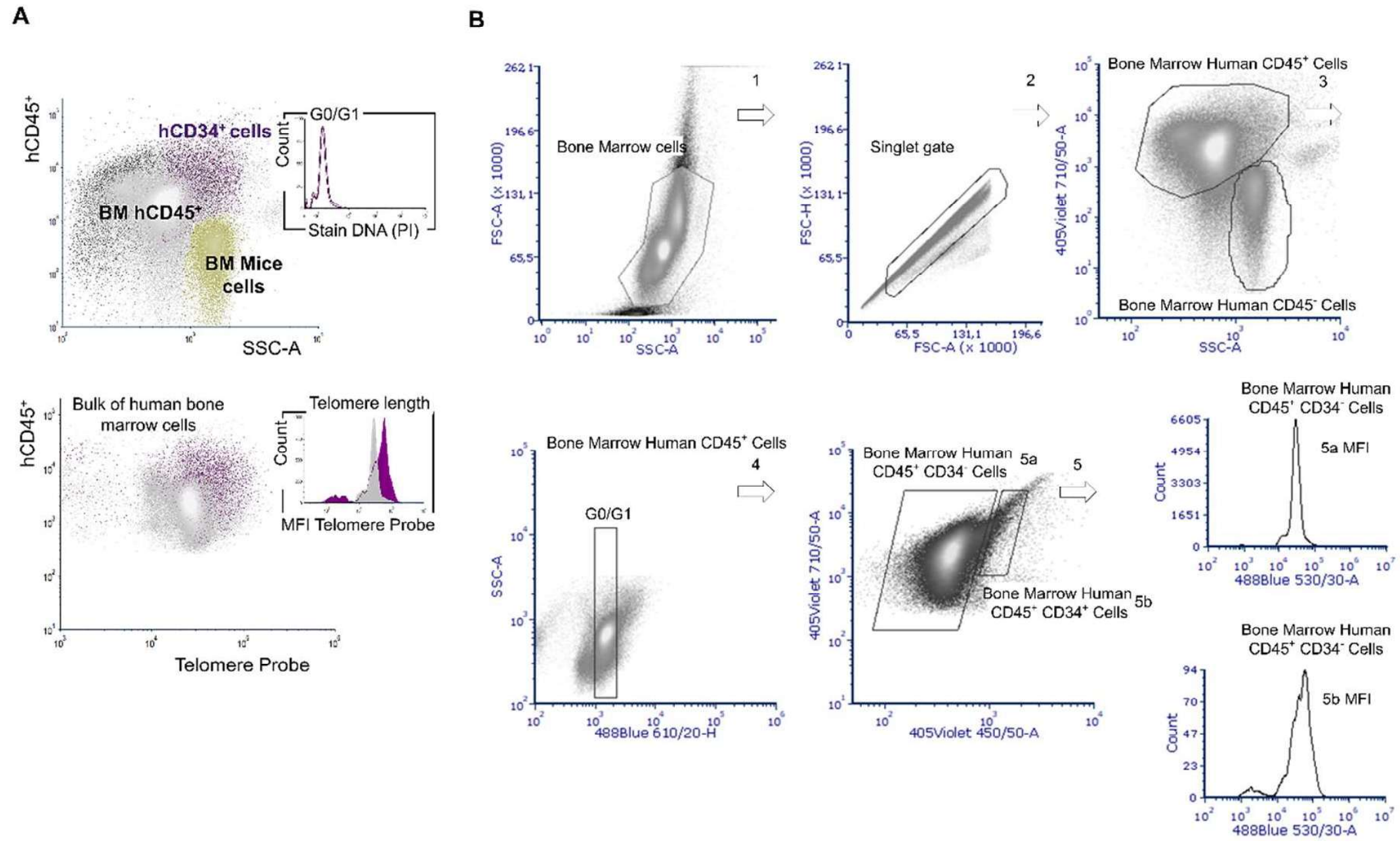

Fig. S5. Flow-FISH gating strategy for telomere length analysis of engrafted human hematopoietic cells in mouse bone marrow. (A) Flow-FISH analysis of telomere length in engrafted human cells (representative dot plots). Top: Flow cytometry gating of human and mouse cells, showing human $CD45^+$ cells (grey), human $CD45^+CD34^+$ cells (purple), and residual mouse cells (ochre) on an hCD45 versus side scatter (SSC) plot. G_0/G_1 cell-cycle phases are indicated for the human cell populations. Bottom: Telomeric probe fluorescence profiles in human $CD45^+CD34^-$ and $CD45^+CD34^+$ cells, shown as overlays of mean fluorescence intensity (MFI). (B) Gating strategy used for Flow-FISH–based telomere length assessment in human hematopoietic cells engrafted in NSG mice. Sequential gating steps are indicated by numbers in the dot plots. Human cells were identified using CD45 and CD34 monoclonal antibodies conjugated to Brilliant Violet 711 and Brilliant Violet 421, respectively. G_0/G_1 cell-cycle phases were discriminated using propidium iodide as a DNA

content marker. Following gating, telomere probe MFI was quantified in the $CD45^+CD34^-$ (population 5a) and $CD45^+CD34^+$ (population 5b) compartments, as shown in dot plot 5.

Table S1. Pre-rRNA Species Detected by Specific Maturation Amplicons

**Pre-rRNA fraction (amplicons):**	**Northern Blot detected pre-rRNA species**											
	47S	45S	43S	41S	34S	32S	30S	26S	21S	21S-C	18S-E	12S
5´ETS-18S	X	X	X		X		X	X				
18S- ITS1	X	X	X	X	X		X	X	X	X		
5,8S - 5´ITS2	X	X	X	X		X						X
3´ITS2-28S	X	X	X	X		X						
*41S, 21S-21SC				X*					X*	X*		

*41S, 21S-21SC fraction is calculated as the difference between fractions (18S- ITS1)- 5´ETS-18S.

Table S2: Description of the oligonucleotides used in the study

Oligonucleotide	Sequence (5' → 3')	Detection
Psi Fw	CAGGACTCGGCTTGCTGAAG	Psi
Psi Rv	TCCCCCGCTTAATACTGACG	Psi
Alb Fw	GCTGTCATCTCTTGTGGGCTG	Albumin
Alb Rv	ACTCATGGGAGCTGCTGGTTC	Albumin
seqDKC1Fw	GATGTAGCCGTGAGTAGTAATGTGTTTG	Genomic human DKC1
seqDKC1Rw	CAATCCCCACATACTCTTTGCCTG	Genomic human DKC1
DKC1 Model Fw	TGTTTTGTGTTGTGTCGTTGCTT	DKC1 intron 3
DKC1 Model Rv	TTGCACAGTGCCTCCTCCC	DKC1 intron 4
DKC1 Control Fw	TTCCAGGACAGGTTTCATTAATCTT	DKC1 intron 4-exon5
DKC1 Control Rv	CCGAAGTATCCGTCGAATCC	DKC1 exon 5
HBB Fw	GCTTCTGACACAACTGTGTTCACTAGC	HBB
HBB Rv	CACCAACTTCATCCACGTTCACC	HBB
Telomere Fw	CGGTTTGTTTGGGTTTGGGTTTGGGTTTGGGTT TGGGTT	Telomere
Telomere Rv	GGCTTGCCTTACCCTTACCCTTACCCTTACCCT TACCCT	Telomere
DKC1 Fw	AAGCCCCGCAGGTAGTTGCC	DKC1 cDNA
DKC1 Rv	CTCGGCCCCGCTCTCCAGAC	DKC1 cDNA
TERC Fw	GCGCGCTGTTTTTCTCGCTGAC	TERC cDNA
TERC Rv	TAGAATGAACGGTGGAAGGCGGC	TERC cDNA
TERT Fw	AACATGGACTACGTCGTGGGAGC	TERT cDNA
TERT Rv	ACGCTGAACAGTGCCTTCACCC	TERT cDNA
GAPDH Fw	AACGGATTTGGTCGTATTGG	GAPDH cDNA
GAPDH Rv	TGGGTGGAATCATATTGGAAC	GAPDH cDNA
CDKN1A Fw	GCTCTGCTGCAGGGGACAGC	CDKN1A cDNA
CDKN1A Rv	TCAGCCGGCGTTTGGAGTGG	CDKN1A cDNA
MYC Fw	TGCTCCATGAGGAGACACCGC	MYC cDNA
MYC Rv	TTGCCAGGAGCCTGCCTCTTTTC	MYC cDNA
RTEL1 Fw	CCGCGGGCTGTAGCCAACTC	RTEL1 cDNA
RTEL1 Rv	TCTCCTCCTGGGGTCCCGGT	RTEL1 cDNA
POT1 Fw	AGGAGACTCAGGGTATCACCAGC	POT cDNA
POT1 Rv	AGTGCGAGGTATGATAGGGGCTC	POT cDNA

CEBPA Fw	CAAGAGCCGCGACAAGGCCA	CEBPA cDNA
CEBPA Rv	GAAGATGCCCCGCAGCGTGT	CEBPA cDNA
TRF1 Fw	ACCTGTTTCAAAGAGTCAGCCGGT	TRF1 cDNA
TRF1 Rv	CCCTCTCCATATTTCCTCACGCCA	TRF1 cDNA
TRF2 Fw	AGGAGACTTGGGTGGAAGAGGATGA	TRF2 cDNA
TRF2 Rv	TCCAGCCTTGACCCACTCGCT	TRF2 cDNA
CCNB1 Fw	TGGTGCCAGTGCCAGTGTCTG	CCNB1 cDNA
CCNB1 Rv	TTCCATTGGGCTTGGAGAGGCAG	CCNB1 cDNA
CDK2 Fw	TCATCGGGTCCTCCACCGAGAC	CDK2 cDNA
CDK2 Rv	ACCACAGGGTCACCACCTCATGG	CDK2 cDNA
TZAP Fw	GGAACAGGTCTTCACGTGCTCTG	TZAP cDNA
TZAP Rv	GCAGGAGGAACACTTGTAGGGC	TZAP cDNA
5´ETS-18S Fw	CGTCCGTCCGTCGTCCTCCT	5´ETS rRNA
5´ETS-18S Rv	ACTGGCAGGATCAACCAGGTAGGTA	18S rRNA
18S-ITS1 Fw	AGGATCATTAACGGAGCCCGGAGG	18S rRNA
18S-ITS1 Rv	GAGCGAACGAACGGGCACGC	ITS1 rRNA
18S Fw	GTGACCACGGGTGACGGGGA	18S rRNA
18S Rv	TACCTCCCCGGGTCGGGAGT	18S rRNA
5,8S Fw	GCGGTGGATCACTCGGCTCG	5,8S rRNA
5,8S Rv	CGTAGCCCCGGGAGGAACCC	5,8S rRNA
5,8S-5’ITS2 Fw	TGTCTGAGCGTCGCTTGCCG	5,8S rRNA
5,8S-5’ITS2 Rv	TGCGCTTAGGGGGACGGAGG	5’ITS2 rRNA
3’ITS2-28S Fw	TGTCTGAGCGTCGCTTGCCG	3’ITS2 rRNA
3’ITS2-28S Rv	TGAGGTCGCGTCTCGGAGGG	28S rRNA
28S Fw	TGCGGACCAGGGGAATCCGA	28S rRNA
28S Rv	CGCCGTTTACCCGCGCTTCA	28S rRNA
GAPDH Fw	CCCCTCTGCTGATGCCCCCA	GAPDH cDNA
GAPDH Rv	TGACCTTGGCCAGGGGTGCT	GAPDH cDNA

Oligonucleotides used in this study (sequences are given 5′→3′). Each oligonucleotide is listed with its sequence and the target or purpose for which it was designed (e.g., *Psi*, *Albumin*, *DKC1* genomic locus, *HBB*, etc.).

Table S3. Description of the different taqman probes used in the study amplicons

Taqman probe	sequence	Fluorochrome/quencher
Psi	CGCACGGCAAGAGGCGAGG	FAM/BHQ1
Alb	CCTGTCATGCCCACACAAATCTCTCC	VIC/BHQ1
DKC1WT probe	ACAACACACTATACACCTCTTGC	HEX/BHQ1
DKC1X-DC probe	AGGGCGACACACTATACACCT	FAM/BHQ1
DKC1Control probe	ACAAGCCCTCTAACCCCTCTTCC	5Cy5/BHQ2

Sequences and labels of TaqMan probes used in this study. Each probe's DNA sequence is shown along with its fluorescent reporter and quencher (e.g., FAM/BHQ1, VIC/BHQ1, HEX/BHQ1, Cy5/BHQ2), corresponding to the specific qPCR/ddPCR amplicons in the assay.
